# A multi-omics approach to Epstein-Barr virus immortalization of B-cells reveals EBNA1 chromatin pioneering activities targeting nucleotide metabolism

**DOI:** 10.1371/journal.ppat.1009208

**Published:** 2021-01-26

**Authors:** R. Jason Lamontagne, Samantha S. Soldan, Chenhe Su, Andreas Wiedmer, Kyoung Jae Won, Fang Lu, Aaron R. Goldman, Jayamanna Wickramasinghe, Hsin-Yao Tang, David W. Speicher, Louise Showe, Andrew V. Kossenkov, Paul M. Lieberman

**Affiliations:** 1 The Wistar Institute, Philadelphia, Pennsylvania, United States of America; 2 Biotech Research and Innovation Centre (BRIC), University of Copenhagen, Copenhagen, Denmark; Brigham and Women's Hospital, UNITED STATES

## Abstract

Epstein-Barr virus (EBV) immortalizes resting B-lymphocytes through a highly orchestrated reprogramming of host chromatin structure, transcription and metabolism. Here, we use a multi-omics-based approach to investigate these underlying mechanisms. ATAC-seq analysis of cellular chromatin showed that EBV alters over a third of accessible chromatin during the infection time course, with many of these sites overlapping transcription factors such as PU.1, Interferon Regulatory Factors (IRFs), and CTCF. Integration of RNA-seq analysis identified a complex transcriptional response and associations with EBV nuclear antigens (EBNAs). Focusing on EBNA1 revealed enhancer-binding activity at gene targets involved in nucleotide metabolism, supported by metabolomic analysis which indicated that adenosine and purine metabolism are significantly altered by EBV immortalization. We further validated that adenosine deaminase (ADA) is a direct and critical target of the EBV-directed immortalization process. These findings reveal that purine metabolism and ADA may be useful therapeutic targets for EBV-driven lymphoid cancers.

## Author summary

EBV immortalization recapitulates aspects of natural B-cell development from germinal center reaction to memory B-cell differentiation. Here, we provide an integrated multi-omic approach to investigating the EBV-induced changes in B-cell transcriptome using RNA-seq, epigenome by ATAC-seq, and metabolome by mass spectrometry of polar metabolites. We identify purine metabolism as a major pathway that is significantly altered by EBV in each data set. We focus on EBNA1 and find that it binds directly to enhancer elements in a subset of genes, including key regulators of purine metabolism such as adenosine deaminase (ADA) and adenylate kinase 4 (AK4). EBV immortalized B-cells are strongly dependent on ADA for proliferation and inhibitors of ADA prevent B-cell immortalization by EBV. These findings identify purine metabolism as a major pathway targeted by EBV during primary infection and suggest that EBNA1 functions as a pioneering transcription factor that reprograms key regulatory genes in purine metabolism pathway.

## Introduction

EBV is a human tumor virus responsible for multiple forms of B-cell lymphomas, as well as NK/T cell lymphoma, ~10% of all gastric carcinomas, and the large majority of undifferentiated nasopharyngeal carcinomas (NPC) [1,2]. In total, EBV-associated tumors account for ~1–2% of all human cancers [3]. EBV has also been implicated in a range of autoimmune diseases, including multiple sclerosis [4]. With greater than 90% of the global population infected with EBV, understanding the biology of the virus and the cellular impact of infection is important for determining the underlying mechanisms involved in viral oncogenesis.

EBV primarily infects resting B-lymphocytes, where it eventually establishes a latent infection as the infected cell undergoes a virus-induced transition towards a memory B-cell. This EBV-induced transition occurs as the virus mimics the natural pathways of antigen-induced B-cell maturation and germinal center reaction, with hyper-proliferation followed by formation of long-lived memory B-cells [5,6]. As a consequence of hijacking these maturation pathways, EBV has the hallmark characteristic of immortalizing primary B-cells infected *in vitro*, establishing latently infected lymphoblastoid cell lines (LCLs). These LCLs serve as an important model for understanding the initial steps of infection, the basic biology of EBV-induced B-cell maturation, and the potential mechanisms of EBV oncogenesis.

EBV-mediated immortalization of B-cells follows a stepwise cascade of events, typically broken down into distinct functional phases (reviewed in [7]). The initial phase, referred to as pre-latency, begins within hours after infection and involves the expansion of individual infected cells and extensive remodeling of the cellular transcriptome as these cells prepare to replicate [8–10]. Between day 3 and 4 post-infection, cells undergo their first round of replication [8] and proceed into a period of hyperproliferation in which cells divide as often as every 8-12hrs. During these early stages there is transient expression of viral lytic genes, despite a lack of lytic replication [11]. There is also significant activation of DNA stress response pathways, particularly in response to the replicative stress associated with hyperproliferation and insufficient nucleotide pools [12–15]. Over time, cells which stabilize their replication and escape stress-induced growth arrest, typically by day ~21 post-infection, are immortalized and become long-lived B-cells which maintain the viral genome as an episome tethered to the host chromatin.

This EBV-mediated immortalization cascade, and associated reprogramming of the cellular transcriptome, is regulated by tightly controlled expression of EBV-encoded proteins and regulatory RNAs, such as EBV nuclear antigen (EBNA) proteins, latent membrane proteins (LMPs), and viral microRNAs. The roles of EBNA2, -3A, -3C, and -LP have been investigated in detail for their functions during immortalization, where they have been shown to cooperate with cellular transcription factors to form viral super-enhancers [16–18]. The viral protein EBNA1, on the other hand, is less well understood for its role during primary immortalization. EBNA1 is essential for maintenance of the EBV episome during latent infection in proliferating cells, and as such, is the only viral protein found in all EBV-associated malignancies and latency-types (reviewed in [19,20]). Studies have shown that EBNA1 is critical for B-cell immortalization, in part through transcriptional activation of EBNA2 [9,21] and that EBNA1 binds with high-affinity and specificity to several cellular loci which are also implicated in EBNA1-dependent transcriptional regulation, including IL6R, MEF2B, and EBF1 [22]. The potential for EBNA1 as a transcriptional regulator is also seen in the fact that EBNA1 transactivates several viral genes during latency [23–25] and contributes directly or indirectly to the regulation of many cellular genes [26–28].

In this study, we utilize a multi-omics approach to identify changes to the cellular epigenome that correlate with transcriptional and metabolic changes during the EBV immortalization cascade. We identify changes in cellular chromatin structure and accessibility using the Assay for Transposase Accessible Chromatin using sequencing (ATAC-seq) [29,30]. We correlate this ATAC-seq data with known datasets of cellular and viral transcription factor binding sites and epigenetic marks, and further analyze these for gene expression changes using concurrent RNA-seq analysis. We focus on the direct DNA binding properties of EBNA1 to identify a category of differentially regulated genes in the nucleotide metabolism pathway, and further substantiate these changes through global metabolomic analysis. We identify adenosine deaminase (ADA), a central regulator of purine metabolism, as a direct target of EBNA1 to show that EBV targets a central regulator of purine metabolism and factor critical for B-cell survival and functionality. Taken together, these findings provide new insight into the role of EBV in coordinating epigenetic, transcriptomic and metabolic reprogramming of B-cells during the immortalization process.

## Results

### Integrated-omics analysis of B-cell immortalization by EBV

**Fig 1:**
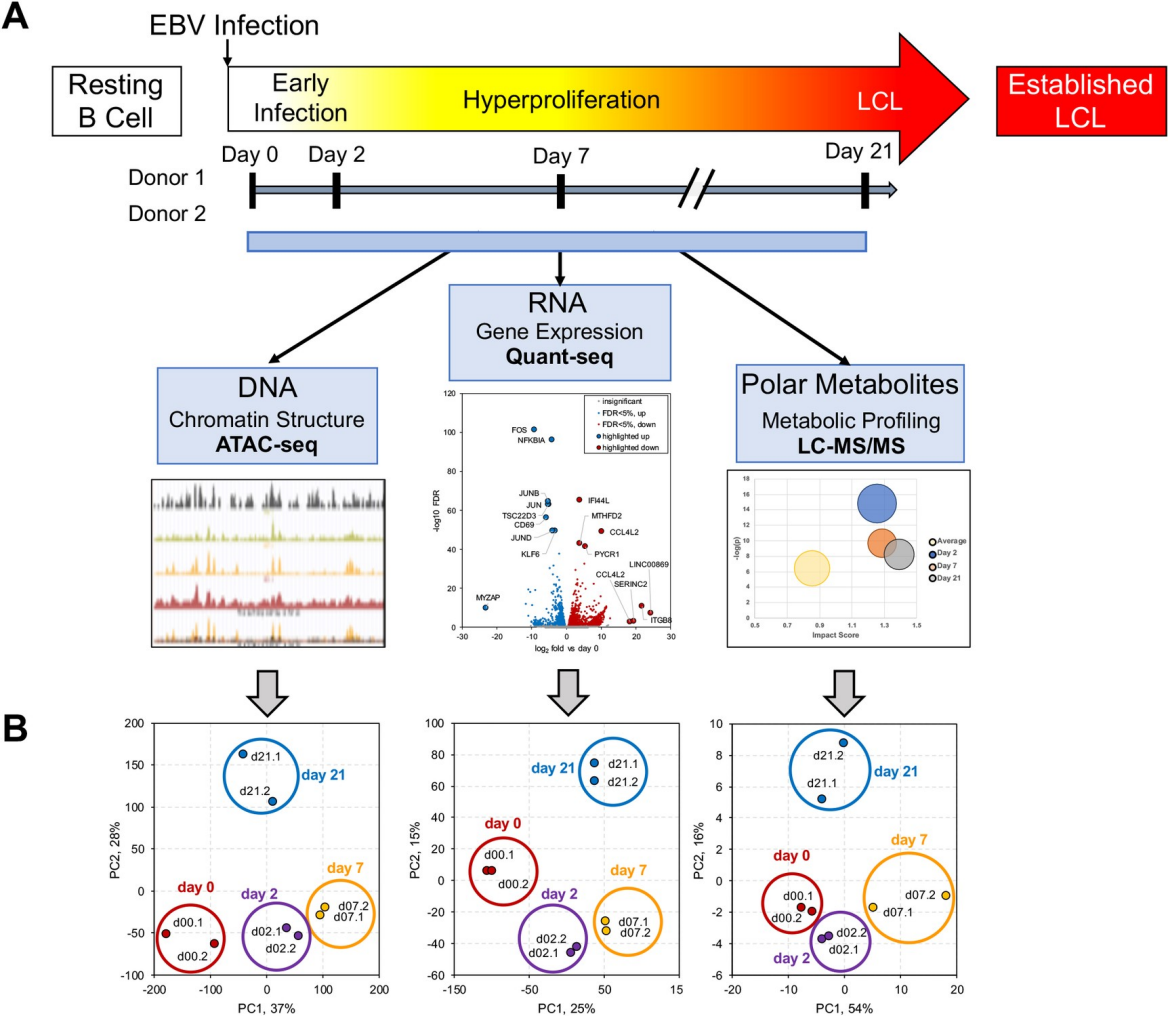
Overview of Integrated Omics Analysis of EBV immortalization of B-lymphocytes. (A) Schematic representation of multi-omics experimental design across the time course of EBV-mediated B-cell immortalization. (B) Independent principal component analyses for ATAC-seq, RNA-seq, and metabolomics datasets.

We investigated EBV-mediated changes to the host cell during primary B-cell immortalization by establishing an integrated dataset of chromatin accessibility, transcription, and metabolic data (Fig 1). Changes in host cell chromatin accessibility were determined using ATAC-seq, and these observations were concurrently supported phenotypically with transcriptome and metabolomic profiling (Fig 1A). To assess changes throughout the immortalization timeline, uninfected samples from Day 0, infected cells in the pre-latent stage at Day 2, hyperproliferating cells at Day 7, and early LCL cells at Day 21 were used. Importantly, all three independent datasets show a similar pattern of sample distribution by principal component analysis, confirming the reliability of the sample sets across the analysis platforms (Fig 1B).

### Changes in chromatin accessibility during EBV induced B-cell immortalization

**Fig 2:**
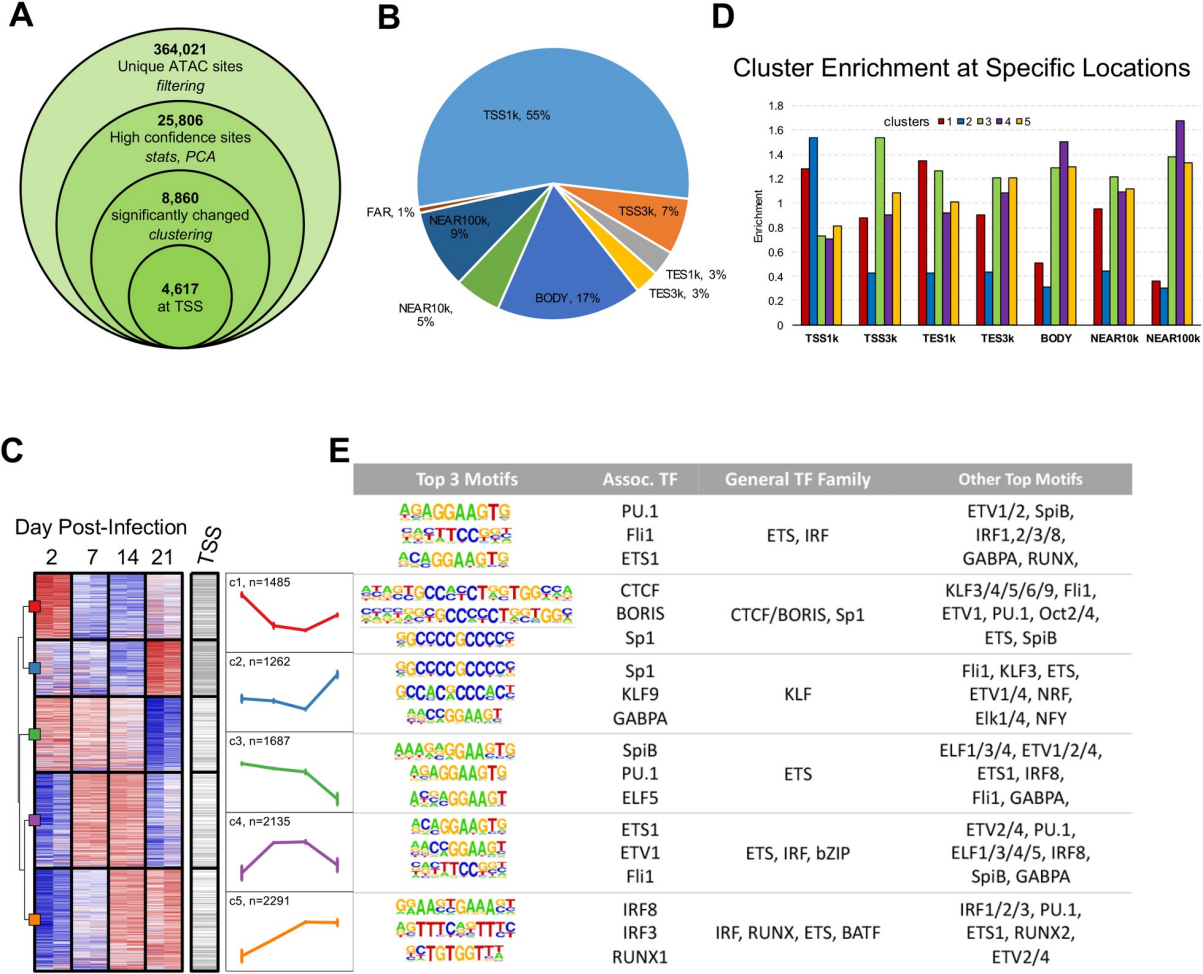
ATAC-seq analysis of accessible chromatin across EBV immortalization. Chromatin accessibility was determined over the infection time course using ATAC-seq. (A) Number of ATAC-seq sites at different stages of data filtering and analysis of differentially accessible chromatin. (B) Distribution of ATAC-seq sites relative to genes. (C) Heatmap showing distribution of ATAC-seq signal across time and unsupervised clustering for all differentially accessible ATAC-seq peaks at any time point. ATAC-seq sites within 1kb from any transcription start site (TSS) are indicated. Mean signal profile for each cluster was plotted to visualize overall pattern of changes. (D) Enrichment of ATAC-seq sites within each cluster at defined genomic locations, calculated as ratio between percent of sites within the region from the cluster vs all ATAC-seq sites. (E) DNA binding motif analysis of peaks within each ATAC-seq cluster. Logos and factor names for the top 3 motifs for all ATAC-seq sites and each cluster are shown. Additional transcription factors with significantly enriched motifs are also listed.

To establish a profile of changing chromatin structure, we first assessed the overall structure of the entire ATAC-seq dataset. Upon initial analysis, 364,021 unique ATAC peaks were identified across the dataset, which were filtered down to 25,806 high confidence peaks (Fig 2A). Over 60% of these high confidence peaks were found within 3kb of a transcription start site (TSS), with an additional 17% found along a gene body and 6% within 3kb of a transcription end site (TES). Interestingly, 14% of total peaks were associated with a distal region of the genome, with 5% within 10kb of the gene, and an additional 9% within 100kb (Fig 2B).

To begin to stratify changes in chromatin structure induced in the context of EBV infection we performed unsupervised clustering on 8,860 sites with differentially accessible chromatin, as shown by significantly changed (FDR < 5%) ATAC-seq signal (Fig 2A and 2C). We found that 25% of the significantly changed peaks increased in accessibility within the first 2 days, while 17% decreased during this same time point. Changes in ATAC-seq peaks could be categorized into several clusters. Cluster 1 shows areas of the genome which appear to be closing immediately after infection before ultimately trending back towards a baseline between Day 7 and Day 21, while cluster 4 shows opening immediately after infection before decreasing accessibility as proliferation plateaus after Day 7. Clusters 2 and 3 also show clear opposing regulation, in which the DNA remains relatively stable in both clusters before either opening (cluster 2) or closing (cluster 3) after the hyperproliferation phase. Cluster 5 suggests that the largest group of changing peaks undergo significant opening prior to the hyperproliferation phase, but ultimately plateau in a fashion similar to the proliferative rate of infected cells (Fig 2C). The genomic distribution of the five ATAC-seq clusters was also determined, with clusters 1 and 2 both showing clear enrichment for peaks near the TSS, while clusters 3, 4, and 5 all showed enrichment at sites distal to a TSS (Fig 2D).

### Transcription factor regulation of chromatin structure

To identify cellular transcription factors (TFs) associated with areas of differential chromatin accessibility, we first performed a DNA motif analysis on peaks within each of the five ATAC-seq clusters. As expected, TFs known to function in B-cell activation and proliferation were among the most frequently predicted binding motifs. These included the ETS family motifs and, in particular, the SPI subfamily (including PU.1), across the entire ATAC-seq dataset. Predicted TF binding sites also included multiple interferon response factor (IRF) binding sites within the clusters that open immediately upon infection. Another interesting observation was the identification of CTCF/BORIS sites within cluster 1, which was the only cluster in which Day 0 had the highest signal for all ATAC peaks, implying a significant closing of the DNA immediately after infection (Figs 2E and S1).

**Fig 3:**
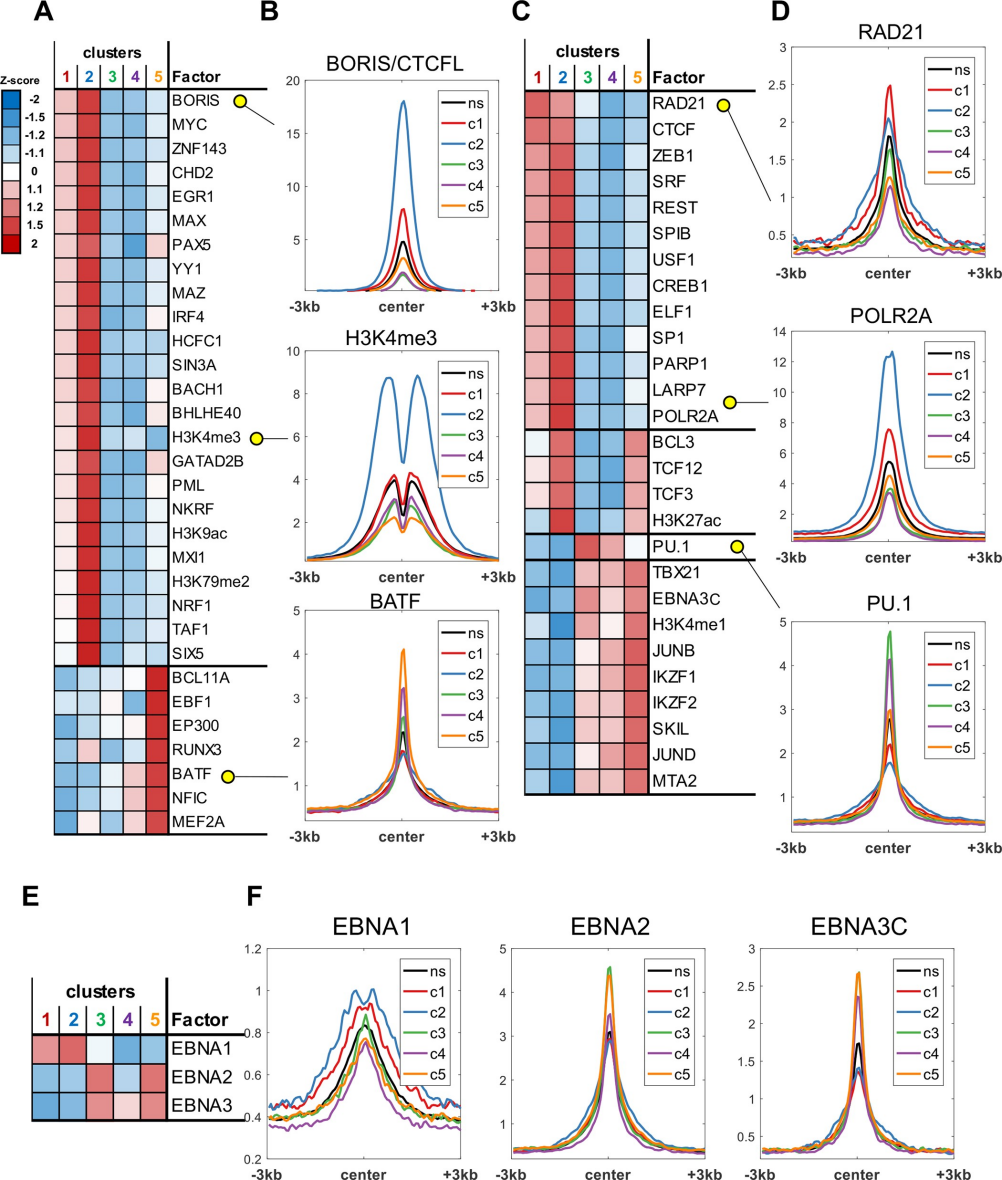
Transcription factor-mediated regulation of chromatin accessibility. Publicly available LCL ChIP-seq datasets were used to derive average binding signal of various factors and histones within high confidence ATAC-seq regions. Heatmaps visualize distribution of mean ChIP-seq signal across clusters. Line plots show average signal profiles for each cluster (c1-c5) as well as non-significantly (ns) changed regions for select specific examples. Examples of factors with higher binding within a single cluster (A) and associated line plots (B) are shown, as well as examples of factors with increased binding in multiple clusters (C, D). (E-F) Distribution of ChIP-seq signal across ATAC-seq clusters for EBNA1, EBNA2, and EBNA3C.

Areas of differential chromatin accessibility were also analyzed for overlap with published ChIP-seq datasets, including TFs, histone modifications and epigenetic modifiers (Fig 3). As with the motif prediction (Fig 2E), we saw significant overlap between areas of differential chromatin accessibility and TFs associated with B-cell signaling and activation (Fig 3A–3D). Interestingly, a number of TF had strong enrichment for ATAC cluster 2, which opens dramatically between Day 7 and Day 21. This cluster is also very strongly enriched for ATAC peaks within 1kb of the TSS, and clearly depleted at other genomic locations, implying these transcription factors are likely regulating chromatin structure directly at the TSS, or proximal promoter regions, compared to other clusters with more distal interactions (Fig 2D). Histone modifications H3K9ac and H3K4me3, indicative of activated promoter regions [31], and RNA polymerase II, indicative of active transcription, also have high enrichment for this cluster of ATAC peaks. Alternatively, the histone mark H3K4me1, associated with enhancer regions [32], is enriched in clusters 3, 4, and 5. All of these clusters are enriched for distal genomic regions, particularly compared to clusters 1 and 2, implying TFs, such as JunD, within these clusters are likely functioning at enhancer regions.

To begin to place EBV regulatory factors into this process, we analyzed the ChIP-seq patterns of EBNA1, EBNA2, and EBNA3C with changing ATAC-seq (Fig 3E and 3F). EBNA2 showed the highest correlation across all changing ATAC-seq peaks, with particular enrichment in ATAC clusters 3 and 5. Similarly, EBNA3C peaks were enriched in ATAC clusters 3–5, while EBNA1 binding was associated with clusters 1 and 2. These findings suggest that EBNAs have non-redundant and temporally separated functions in host-chromatin remodeling during B-cell immortalization, particularly in the context of the differing genomic enrichment of these clusters of differentially accessible regions.

### Transcriptome changes during B-cell immortalization

**Fig 4:**
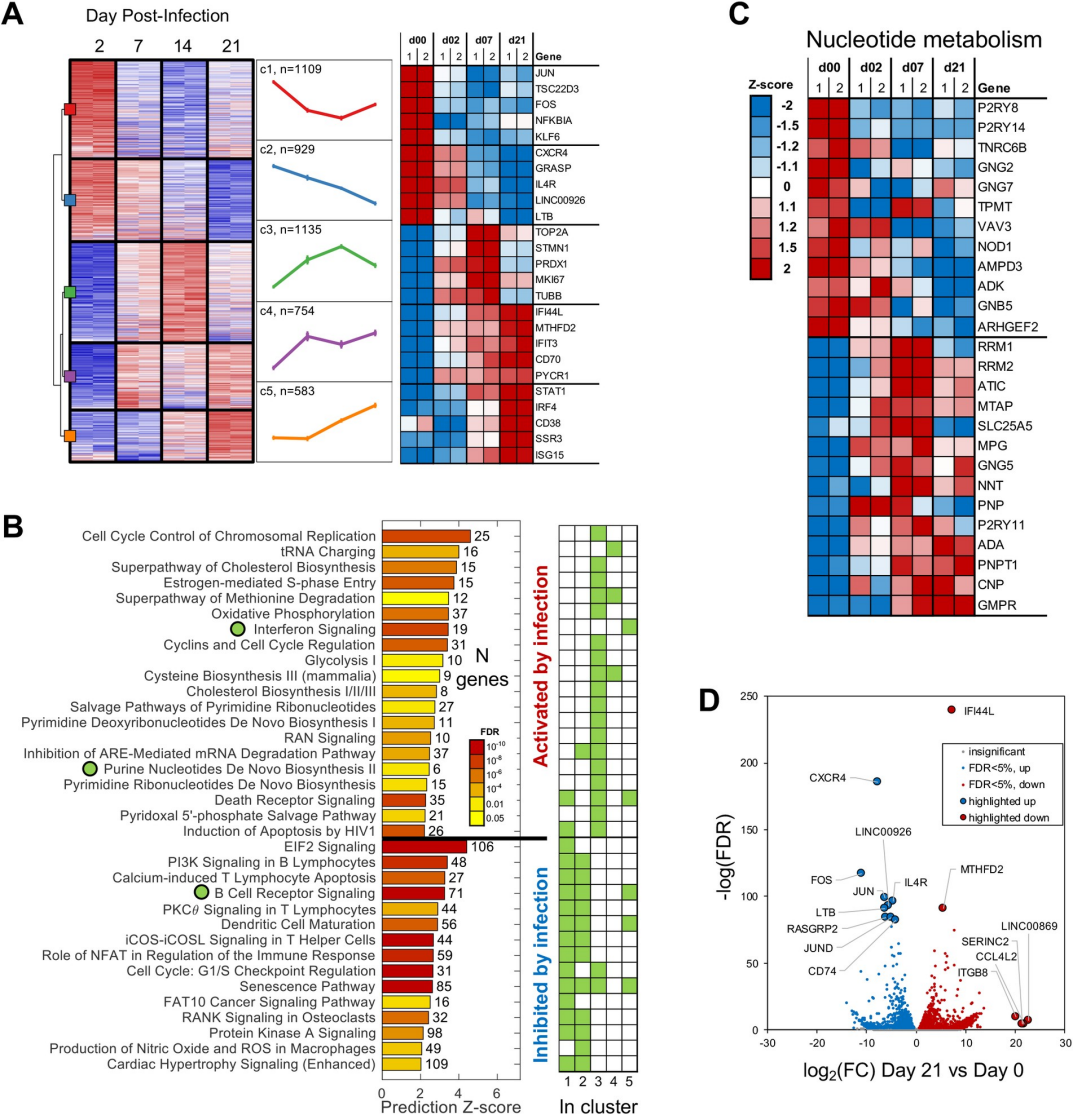
Transcriptomic analysis of EBV-mediated B-cell reprogramming. Analysis of gene expression changes over the time course of infection was performed by RNA-seq and differentially expressed genes at any time point were identified. (A) Unsupervised clustering of differentially expressed genes with representative mean expression pattern is shown for each cluster. The top 5 most changed genes along with their respective relative expression levels are also shown for each cluster. (B) Pathways significantly enriched among all differentially expressed genes. Pathways significantly enriched within specific gene clusters are indicated. (C) Relative expression level of genes associated with the nucleotide metabolism pathway. (D) Volcano plot showing differentially expressed genes at Day 21 post infection with examples of genes continuously differentially expressed across the time course highlighted.

To understand the functional significance of EBV-mediated chromatin remodeling, we performed both transcriptomic and metabolomic profiling concurrently with our ATAC-seq experiments. We first analyzed the RNA-seq dataset (S2C–S2E Fig) with unbiased clustering of all significantly changing genes (FDR < 5%), independent of ATAC-seq data. Five distinct clusters of changing gene expression were identified (Fig 4A), and significant disruption of multiple cellular pathways was identified by pathway analysis of these changing genes (Fig 4B). For example, significant disruption of interferon signaling was identified, with associated genes enriched within RNA cluster 5. This cluster shows continual increased expression after Day 2 post infection, suggesting clear activation of interferon signaling during the immortalization time course (Figs 4B and S2A). In addition, multiple metabolic pathways, including nucleotide and cholesterol metabolism, were disrupted. Genes within these pathways were enriched in RNA cluster 3, which supports a role for these pathways during hyperproliferation as gene expression is immediately increased after infection, before ultimately trending downward as proliferation plateaus by Day 21 (Fig 4C).

In addition to unsupervised clustering, trends within broadly related gene sets were also clearly distinguishable. For example, the expression of many TFs changed significantly over the course of infection, including decreases in *Fos*, *Jun*, and *BCL6*, and increases in *Bach2* and *BATF* (S2B Fig). *CTCFL*, the gene encoding the CTCF-homolog BORIS, is also significantly increased over the course of infection, while *CTCF* itself remains stable (S1D–S1F Fig). In fact, many of the differentially expressed TFs fall out within distinct RNA clusters associated with their expected cellular role suggested by the ATAC-seq dataset. For example, *BATF* (RNA cluster 3) immediately increases expression until it ultimately begins to come down by Day 21. ChIP-seq data suggested that areas of differential chromatin within ATAC cluster 5 were enriched in BATF binding sites, which show continually increased accessibility before plateauing by Day 21, suggesting a functional correlation between levels of BATF and chromatin accessibility at BATF binding sites (Fig 2E). Overall, however, many of the most significantly changed genes were altered immediately after infection and maintained a significantly altered expression level through the course of infection (Figs 4D and S2C).

### Correlation of ATAC-seq and transcriptome profiling

**Fig 5:**
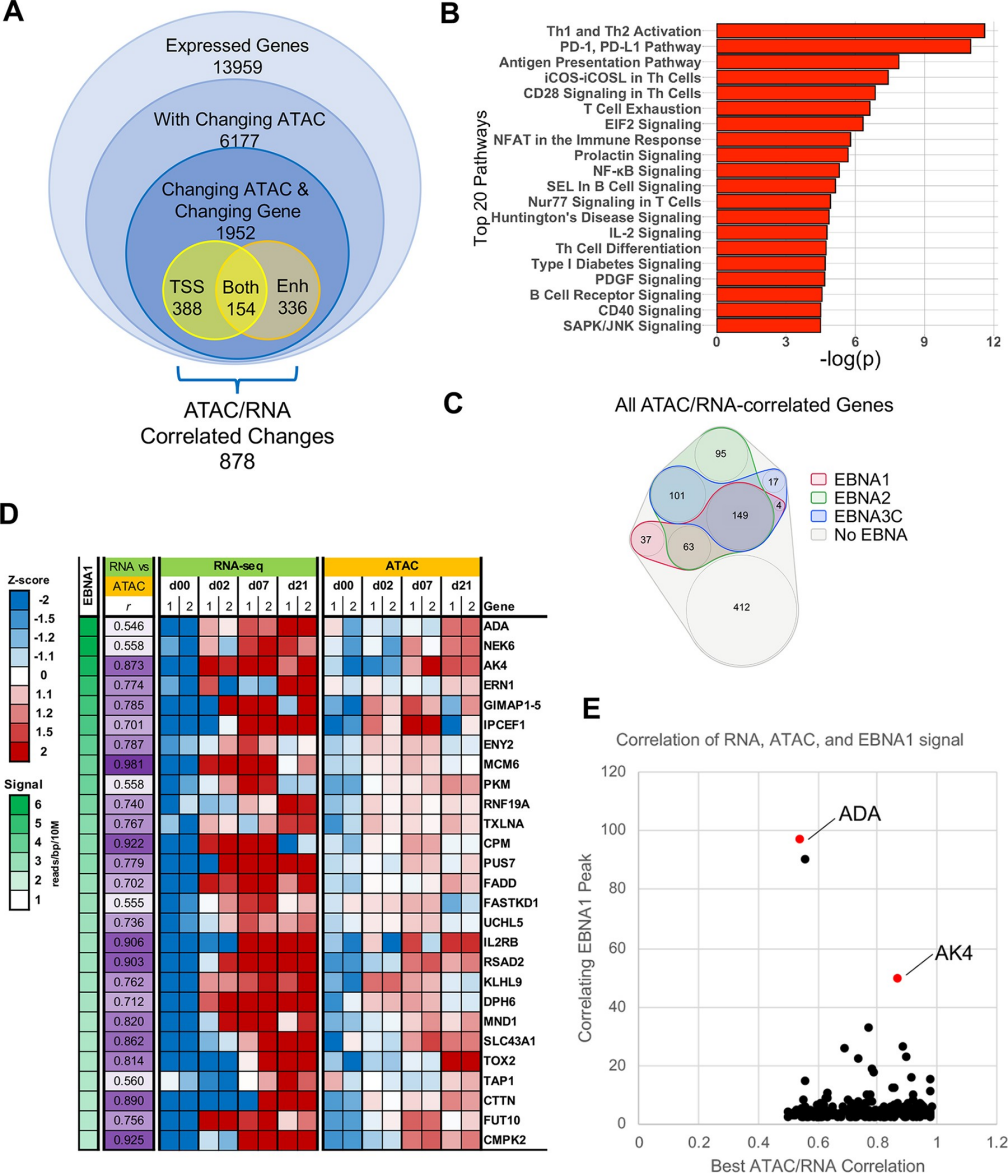
Integration of ATAC-seq and RNA-seq and EBNA ChIP-seq datasets. (A) Transcriptomic data was integrated with ATAC-seq data by generating sequential subsets of genes based on their association with differentially accessibly chromatin, and the associated gene expression pattern. Genes were considered directly correlated if there was correlation (r > 0.5) between changes in gene expression and changes in chromatin accessibility. (B) Pathway analysis was done on the subset of directly correlated genes, with the Top 20 associated pathways shown. (C) Venn diagram indicating the association between directly correlated genes and EBNA1, EBNA2, and EBNA3C DNA binding locations. Genes within the grey cluster show no associated EBNA binding, while those in the colored circles have ChIP-seq peaks for the indicated EBNA protein at the TSS and/or enhancer regions. (D-E) EBNA1-associated directly correlated genes were ordered based on the strength of the associated EBNA1 peak (D), and plotted based on the strength of associated EBNA1 peak versus the strength of the correlation between the RNA expression and ATAC-seq signal patterns (E).

To better define the relationship between the ATAC-seq and transcriptomic datasets, we identified 1952 genes that had significant changes in both RNA expression and ATAC signal, and 878 of those showed a correlation (Pearson r > 0.5) between ATAC-seq signal pattern and RNA expression pattern. The areas of differentially accessible chromatin associated with these 878 genes, referred to as the directly correlated genes, can be broken down by genomic location, and were distributed at both TSS (44%) and at enhancer regions (38%) as called by the GeneHancer database [33]. The remaining 18% showed changing chromatin accessibility at both TSS and an enhancer regions (Fig 5A). While the genes within this directly correlated subset were evenly distributed across the five RNA clusters, they were differentially correlated with the ATAC clusters as genes within specific RNA clusters showed enrichment for particular ATAC clusters (S3 Fig). Pathway analysis of these directly correlated genes showed enrichment in pathways involved in B-and T-cell activation (Fig 5B).

### EBV-mediated regulation of chromatin accessibility

In support of the role of EBV in regulation of the directly correlated gene subset, over 50% of these genes were associated with binding of at least one of the three EBNA proteins. In fact, of the genes that are associated with one of the EBNA proteins, the largest group actually shows binding by all three proteins (Fig 5C). As would be expected, EBNA2 was associated with the largest number of genes in the subset, 408, while EBNA3C was associated with 272 genes and EBNA1 with 253 genes. The high degree of overlap between these factors suggests a mechanism of cooperative regulation in the context of this subset of directly correlated genes, and therefore regulation of pathways involved in the modulation of B-cell signaling and activation (Fig 5B). All three EBNA proteins also show higher than expected binding at both TSS and enhancer sites within this subset of genes (S4 Fig).

### EBNA1-associated regulation

Previous studies have characterized the transcriptional regulatory functions of EBNA2 and EBNA3C [18,34–37]. To further understand the role for EBNA1 in B-cell reprogramming, we analyzed the gene class containing correlated differentially expressed genes and differentially accessible chromatin with EBNA1 ChIP-seq peak intensity (Fig 5D and 5E). Interestingly, 2 of the top 3 genes from this analysis, adenosine deaminase (*ADA*) and adenylate kinase 4 (*AK4*), are involved in different aspects of adenosine metabolism, supporting the previous identification of nucleotide, and specifically purine metabolism, as a significantly EBV-modulated pathway (Fig 4B and 4C).

### Metabolomic profiling identifies purine metabolism as an EBV-regulated pathway

**Fig 6:**
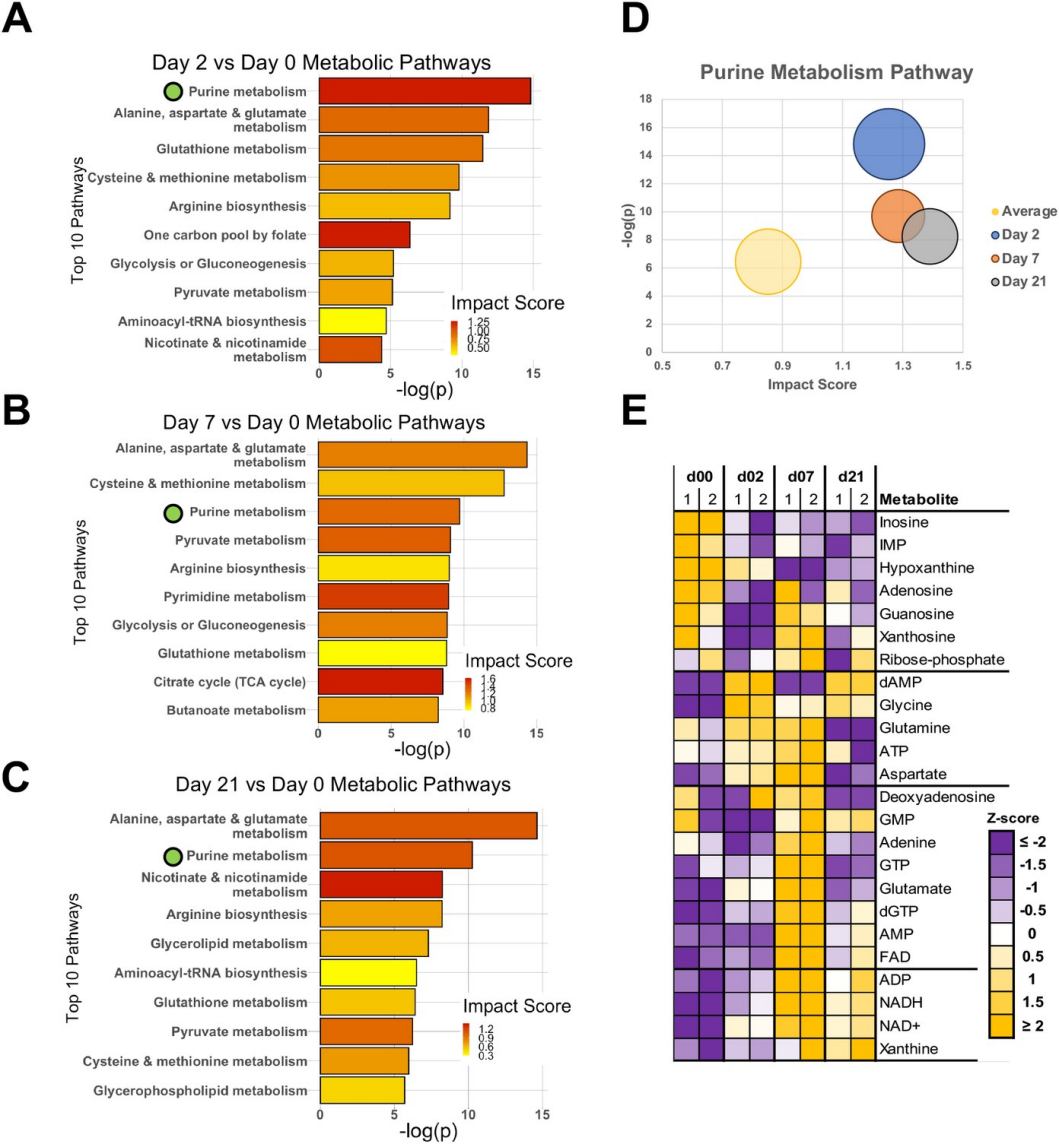
Metabolomic analysis of EBV-mediated B-cell reprogramming. (A-C) Significantly altered metabolites and genes (|FC| >2, FDR < 5%) were used in an integrated pathway analysis to identify altered metabolic pathways at Day 2 (A), Day 7 (B) and Day 21 (C) post-infection. Green marker was used to highlight purine metabolism. (D) Bubble chart showing significant disruption of purine metabolism compared to the average significantly altered pathway. Bubble size represents the ratio of significantly altered metabolites and genes in the pathway compared to the number expected due to chance. (E) Heatmap showing relative levels of metabolites in the purine metabolism pathway. Metabolites are grouped based on general trend of metabolite levels across the complete time course of infection.

Because the transcriptomic analysis identified purine metabolism as a significantly activated pathway during infection and ChIP-seq analysis showed that *ADA* and *AK4* were both highly associated with EBNA1 binding, we used our metabolomic profiling data to determine if specific metabolic pathways were disrupted during the immortalization cascade. Metabolic pathway changes in the context of EBV have been described in other studies [14,38–41], and we confirmed previously EBV-associated metabolic pathways such as fatty acid metabolism and one-carbon metabolism as altered in our dataset (S5 Fig and S2 Table). Because both the gene expression and metabolomic data were generated from the same samples, we utilized an integrated metabolic pathway analysis [42,43] that incorporates significant changes in both gene expression and metabolite levels (|FC| > 2, FDR < 5%). With this approach, significant disruption of multiple metabolic pathways, particularly among nucleotide and amino acid metabolic pathways, was observed across the full-time course (Fig 6A–6C). In looking at these pathways overall, one of the most altered pathways across the entire time course was purine metabolism, as it was one of the three most significantly impacted metabolic pathways in the integrated analysis at all three time points after infection (Fig 6A–6D). Analysis of individual metabolites within the purine metabolism pathway shows distinct patterns indicative of activation of this pathway in response to the need for increased purine nucleotides and increased energetic needs overall during the hyperproliferative phase at Day 7 (Fig 6E).

### EBV-mediated regulation of ADA

**Fig 7:**
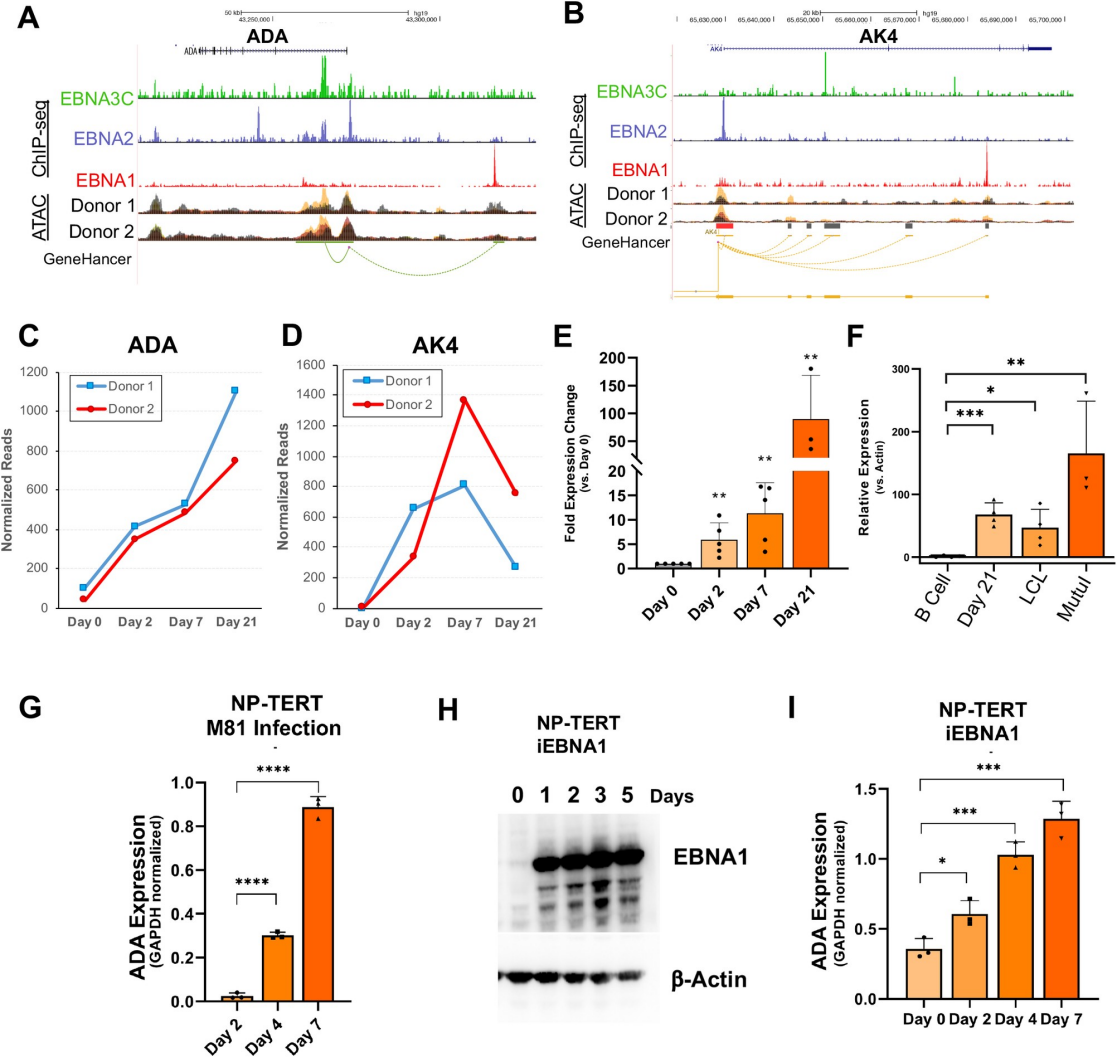
Role of EBNA1 in transcription activation of ADA and AK4 during primary infection. (A-B) Alignment of ATAC-seq data and EBNA1, EBNA2 and EBNA3C ChIP-seq data for ADA (A) and AK4 (B). ATAC-seq data is overlaid for each patient showing Day 0 (black), Day 2 (green), Day 7 (yellow), and Day 21 (red). (C-D) Normalized RNA-seq reads showing expression levels at each time point for ADA (C) and AK4 (D). (E-F) RT-qPCR analysis of ADA levels during primary B-cell infection with EBV (E) or in EBV^+^ cell lines. (G) EBV M81 infection of NP-TERT cells at day 3, 4, or 7 analyzed by RT-PCR for ADA expression relative to GAPDH. (H) NP-TERT cells with Dox-inducible EBNA1 were treated without (0) or with Dox for 1, 2, 3, or 5 days and assayed by Western blot for EBNA1 or loading control β-Actin. (I) iEBNA1 NP-TERT cells were induced with Dox for 0, 2, 4, or 7 days and assayed by RT-qPCR for ADA expression relative to GAPDH. *, p<0.05; ***, p<0.001;****, p < .0001 p values determined by two-tailed t-test; data represents a minimum of 3 independent experiments.

To better understand how EBV, and EBNA1 in particular, may regulate the purine metabolism pathway, we focused on the epigenetic properties of the *ADA* and *AK4* genes, which had previously shown strong EBNA1 binding (Fig 5D and 5E). EBNA1 ChIP-seq revealed strong binding at an enhancer element ~30kb upstream of the *ADA* TSS, and within the body of the *AK4* gene. The major ATAC peak change for *ADA* was near the TSS, and overlapped with EBNA3C, while the TSS was occupied by EBNA2 (Fig 7A). *AK4* also has non-overlapping peaks for EBNA1, EBNA3C, and EBNA2, with the major changing ATAC peak colocalizing at the TSS with EBNA2 (Fig 7B). This EBNA protein-associated differential chromatin accessibility was associated with differential gene expression in the RNA-seq dataset for both *ADA* (Fig 7C) and *AK4* (Fig 7D). To confirm these observations, expression of *ADA* was analyzed by RT-qPCR across multiple primary B-cell infections, with significant increases in the level of *ADA* expression seen at each time point post-infection (Fig 7E). Finally, comparison of the levels of *ADA* across a panel of EBV-positive cell lines shows a significant increase compared to uninfected B-cells (Fig 7F). To determine if EBNA1 alone was sufficient to induce *ADA* expression in EBV negative cell types, we first expressed EBNA1 in EBV negative cell lines BJAB, AKATA, and HEK293T. While transient transfection of EBNA1 led to a ~2 fold increase in *ADA* expression in HEK293T cells (S6 Fig), we were unable to show a significant effect of EBNA1 on *ADA* or *AK4* transcription in BJAB or AKATA cells, likely due to the increased baseline levels of *ADA* in immortalized lymphocytes. To address this, we proceeded to use an alternative model that would allow us to look at both the impact of EBV infection and EBNA1 alone. First, we assayed the effects of EBV infection on tert-immortalized nasopharyngeal cells (NP-TERT) using the lymphoepitheliotropic EBV strain M81. We found that EBV infection of NP-TERT cells resulted in a large (>10 fold) increase in *ADA* by days 4 and 7 post-infection (Fig 7I). To test whether EBNA1 alone was sufficient to activate ADA transcription in the same cell model, we generated an NP-TERT cell line with a doxycycline-inducible EBNA1 gene. In these cells, induction of EBNA1 led to a progressive increase in *ADA* transcription with ~4 fold increase by day 7. Taken together, these findings suggest that EBNA1 contributes to the activation of ADA in some cell types, but may not be sufficient for the complete activation observed during viral infection.

**Fig 8:**
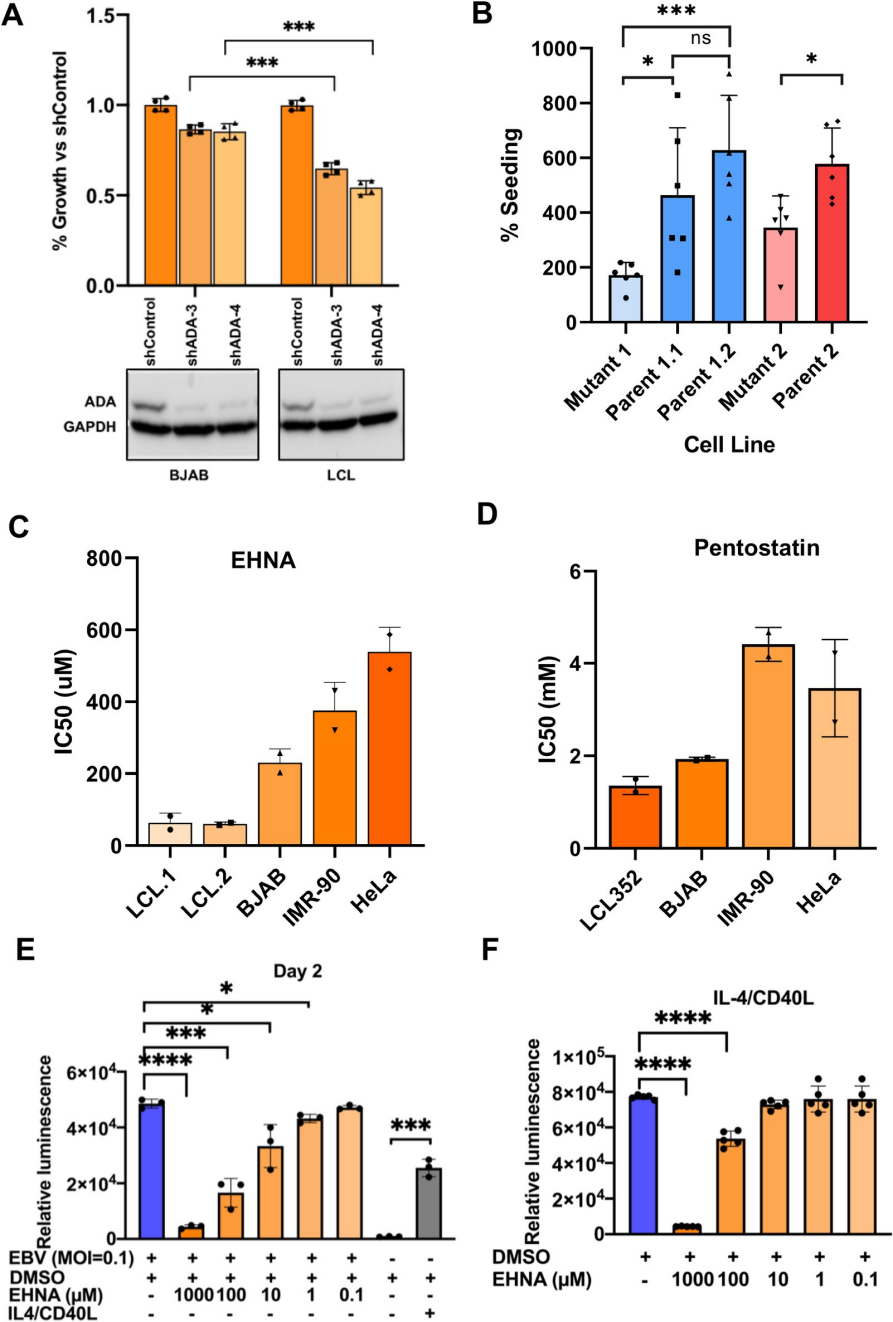
Requirement for ADA activation during EBV immortalization. (A) ADA expression was knocked down by transduction with lentivirus shRNA in EBV^-^ BJAB or LCL cells. Relative proliferation was determined by CellTiter-Glo assay and normalized to shControl-transduced cells. Western blot for ADA and loading control GAPDH (lower panel). (B) Reseeding efficiency was compared between LCLs from ADA-associated SCID patients and their phenotypically normal parents (*, p<0.05; **, p<0.01; ***, p<0.001; ns, not significant; p values determined by two-tailed t-test; data represents a minimum of 3 independent experiments). (C) EBV^+^ and EBV^-^ cell lines were treated with serial dilutions of the ADA inhibitor, EHNA, for 72hrs and the IC50 value for each cell line was determined. Data represents two independent experiments with 3 technical replicates per experiment. (D) Same as in panel C, except using Pentostatin as ADA inhibitor. (E) Primary B-cells infected with EBV in the absence (blue bar) or presence of increasing concentrations of EHNA and assayed at day 2 for ATP levels by CellTiterGlo. In parallel, primary B-cells were treated without or with IL-4/CD40L and assayed with CellTiterGlo. (F) Primary B-cells treated with IL-4/CD40L in the absence or presence of increasing concentrations of EHNA and assayed for ATP levels by CellTiterGlo. *, p<0.05; ***, p<0.001; ****, p < .0001; p values determined by two-tailed t-test; data represents a minimum of 3 independent experiments.

To investigate the functional significance of ADA in EBV infection and B-cell immortalization, we compared the effects of shRNA knockdown of ADA in EBV^+^ LCLs relative to EBV^-^ BJAB cells (Fig 8A). We found that EBV^+^ cells were consistently more sensitive to ADA knockdown than EBV^-^ cells, with a >50% decrease in growth of LCLs after efficient ADA knockdown compared to only a ~10% reduction in growth in BJAB cells (Fig 7G). Knock-down in EBV^+^ BL cells carrying a type I latency pattern showed an intermediate phenotype, with ~30% reduction in cell growth (S7A Fig). We also assayed the growth properties of LCLs derived from patients with genetically confirmed mutations in *ADA* conferring severe combined immunodeficiency syndrome (Fig 8B). LCLs derived from patients with mutant *ADA* had significantly lower growth and re-seeding potential compared to LCLs derived from their phenotypically-normal parents. To investigate the contribution of ADA enzyme activity in EBV biology, we compared the effects of two different ADA enzyme inhibitors on several different EBV positive and negative cell lines (Fig 8C and 8D). We compared two different purine analogue inhibitors of ADA for their ability to affect LCLs compared to EBV negative B-cell and epithelial cell lines. We found that LCLs were significantly more sensitive to EHNA and pentostatin than were the cell lines lacking EBV (Fig 8B and 8C). EBV positive Mutu I cells, which exhibit Type I latency and as such express only EBNA1, were also found to be sensitive to EHNA, but to a lesser extent than LCLs (S8 Fig). We also assayed the ability of EHNA to inhibit primary B-cell proliferation during EBV immortalization (Figs 8E and S9) or after treatment with CD40L/IL4 (Figs 8F and S9). We found that EHNA inhibited both EBV-induced and CD40L/IL4 stimulated B-cell proliferation, but that EBV induced B-cell proliferation was more sensitive to EHNA inhibition. This modest, but statistically significant, effect of EHNA in CD40L/IL4 treated cells was similar to the effect seen on ATP levels and proliferation in CD40L/IL4 treated cells in which ADA had been knocked down by shRNA (S7B Fig). Taken together, these findings indicate that ADA plays an important functional role in EBV-dependent B-cell proliferation.

## Discussion

Identifying the regulatory mechanisms linking the epigenetic, transcriptomic and metabolic reprogramming during EBV-induced B-cell immortalization is important for understanding EBV oncogenesis. Here, we provide a map of EBV-mediated changes to B-cell chromatin accessibility during the early stages of infection and correlate this with transcriptomic and metabolomic outcomes of this epigenetic regulation. Using the ATAC-seq protocol we were able to show significant remodeling of the B-cell chromatin in the context of EBV infection. We correlate these changes with alterations to the host cell transcriptome and metabolome, and positional proximity to viral regulatory factors EBNA1, EBNA2, and EBNA3C.

Previous work has extensively characterized the transcriptomic changes of EBV-induced B-cell immortalization [8,10,44]. Our focus has been on transcriptome modifications centered primarily on its integration with ATAC-seq and metabolomic datasets. Our RNA-seq is consistent with previous reports, and identifies pathways such as interferon [10,35] and B-cell receptor signaling [5], regulation of the cell cycle [8], and metabolic pathways such as cholesterol biosynthesis [39] and one-carbon metabolism [38]. We also see that the largest clusters of genes all show dramatic changes in gene expression between Day 0 and Day 2, supporting previous work suggesting significant reprogramming of the cellular transcriptome occurs prior to the hyperproliferation phase [8].

Correlation analyses allowed us to identify a specific set of genes for which the level of change in chromatin accessibility corresponds to an associated change in gene expression. Transcriptomic data identified many EBV-regulated pathways, including those that are directly correlated and linked to cellular pathways known to be important for activation of B-cell signaling. The ETS-family transcription factors, especially PU.1, were found to be among the most enriched for this category of change in chromatin accessibility and transcriptional activation, and EBNA2 and EBNA3C have been previously found to cooperate with PU.1 at specific genes [45]. Our data supports that this occurs genome-wide for a large class of genes important for B-cell activation. Our findings also reveal a potentially novel role for CTCF and the CTCF-homolog CTCFL (BORIS) at chromatin sites undergoing reduced accessibility and transcriptional silencing. CTCF sites correlated with genes repressed early after infection, while BORIS was associated with ATAC-seq clusters that open later in the immortalization time course, suggesting that BORIS acts as a competitive inhibitor of CTCF [46–48], which has been implicated in EBV-mediated gene regulation and chromosome conformation. The colocalization of EBNA1, 2, and 3C at, or in close proximity to these ATAC-seq changing sites suggests that EBV actively reprograms the chromatin accessibility landscape at many cellular sites throughout the genome. The data suggests that EBV is playing a direct role in the regulation of these correlated genes, as over 50% of the genes are associated with binding of at least one of the EBNA proteins and 17% are associated with all three of the EBNAs analyzed.

Modulation of transcription frequently requires pioneering factors to invade and disrupt developmentally established nucleosome positions and chromatin structures [49–52]. In the context of EBV, chromatin remodeling and pioneer factor activity is likely to play an important role in the regulated changes in gene expression that occur during the early stages of infection and the immortalization cascade. In fact, multiple cellular transcription factors that have been implicated as pioneer transcription factors, including EBF1 [53,54] and PU.1 (Spi1) [55,56], have been shown to interact and colocalize with EBV factors, including EBNA2 and EBNA3C, to regulate many target genes [18,57]. Transcriptomic and epigenetic regulation in B-cell remodeling has been reported for multiple EBV factors, including EBNA2, -3C, and -LP that associate with cellular sequence-specific transcription factors to form higher-order super-enhancers [16,18]. However, far less is known about the epigenetic effects of the sequence-specific DNA binding factor EBNA1. Our findings suggest that EBNA1 can function as a pioneering factor for a subset of cellular genes, which is in line with previous reports suggesting EBNA1 mimics functional and structural characteristics of FoxA, a pioneer transcription factor [58], and the HMGA family of proteins, which function as chromatin remodelers [28].

Focusing on the potential pioneering activity of EBNA1, we were able to identify genes involved in purine metabolism, including *AK4* and *ADA*. The importance of ADA in lymphocyte development is underscored by its frequent mutation in the most common form of severe combined immunodeficiency disease (SCID), and ADA deficiency is particularly detrimental to B-cells, including causing impaired germinal center maturation [59]. Decreased ADA alters the intracellular nucleotide balance and energetic state, as well as altering signaling through the cell surface adenosine receptors, which are vital regulators of an effective and controlled immune response [60–62]. Recent work has also suggested that adenosine may be playing a role in the regulation of the switch between EBV latency and lytic reactivation [63], underscoring the importance of understanding the impact EBV has on ADA and related factors in the context of primary infection. Here, we provide evidence that ADA is a direct target of EBNA1 that may confer pioneering activity during early stages of EBV infection, and that purine metabolism plays a central role in B-cell immortalization. Together, these findings lay the groundwork for pursuing ADA and purine metabolism pathways as targets for more selective therapies to treat EBV-associated lymphoid cancers.

## Materials and methods

### Cell lines

Primary B-cell infections were done in B-cell medium (RPMI supplemented with 20% fetal bovine serum (FBS), 10mM HEPES, 1X Glutamax, and penicillin/streptomycin), and maintained in this medium for a minimum of 4 weeks (or until collected). Once proliferation was stabilized cells were transferred to normal RPMI medium supplemented with 10% FBS and antibiotics. Previously established LCLs, BJAB cells, and Mutu I cells were also maintained in RPMI with 10% FBS and antibiotics. IMR90, HK1, and HEK293T cells (ATCC) were grown as adherent cells in DMEM with 10% FBS, 1x Glutamax, and penicillin/streptomycin. NP-TERT cells (NP460hTert, gift of S. W. [George] Tsao) were grown with EpiLife (MEPI500CA, Gibco) supplemented with K-SFPM and Growth factor (10744019, Gibco). EBNA1 inducible NP-TERT was generated by transduction with pLU-TCMV-FMCS-pPURO expressing Dox-inducible FLAG-EBNA1. LCLs derived from patients with mutant ADA (GM11411, GM02471B) and associated parental lines (GM11412/GM11413, GM02472A) were acquired from the Coriell Institute for Medical Research (Camden, NJ), and maintained as other LCLs.

### Virus and bacmids

Primary infection analyses were done using purified virus from Mutu I cells, a Burkitt’s lymphoma-derived, EBV-positive cell line. To generate virus for infection studies, EBV lytic replication was induced using a combination of sodium butyrate (1μM) and TPA (50ng/ml) for 3 days. Cells and medium were collected and centrifuged twice at 2000 x g for 20mins to remove cellular debris. Cleared supernatants were then passed through a 0.45μM filter and concentrated by ultracentrifugation through a 10% sucrose cushion at 100,000 x g for 1 hour at 4°C. Viral pellets were resuspended in B-cell media (RPMI) and quantified by qPCR comparing copies of EBNA1 vs a standard curve of EBV DNA using Namalwa genomic DNA as standard.

### Isolation of primary B-cells

All infection studies were done with primary B-cells isolated from whole blood based on a modified protocol utilizing lymphocyte separation medium (Lymphoprep, STEMCELL Technologies) and specialized centrifugation tubes (SepMate-50, STEMCELL Technologies). Briefly, whole blood was collected from an individual donor and immediately diluted 1:1 with sterile DPBS supplemented with 2% FBS. Next, 15ml of Lymphoprep was added through the insert hole of SepMate-50 columns, and 30ml of diluted blood was slowly layered over top. Tubes were then spun at 1200 x g for 10 mins at room temperature with full brake (as per manufacturer’s instructions). Supernatant was then transferred to a normal 50ml conical tube, being careful to minimize transfer of the loosely packed erythrocyte layer. Supernatant volume was increased to 50ml with DPBS/2% FBS and spun at 500 x g for 10mins. The supernatant was then removed, and wash was repeated a minimum of 4 additional times. Prior to the final wash, total numbers of peripheral blood mononuclear cells were counted. Final PBMC pellets were then either resuspended at 1x10^8^ cells/ml for freezing and future use or prepared for immediate B-cell purification using the EasySep B-cell immunomagnetic negative selection kit following the manufacturer’s protocol (STEMCELL Technologies). Purified B-cells were resuspended in B-cell medium, counted, and infected immediately after purification.

### EBV infection

For primary infection studies, B-cells were isolated as above and resuspended in 1ml of B-cell medium. Concentrated virus was added at an MOI of 1 and incubated at 37°C with 5% CO_2_ for 3hrs. Cells were then diluted to 0.5 x 10^6^ cells/ml and maintained until collection at the indicated timepoint. For 21 day infections, medium was added at day 10. Infections were monitored by the growth and clumping of cells, a characteristic of lymphoblastoid cell lines.

For all primary B-cell infections, B-cells were not pooled, and instead represent a single infection of B-cells from a single donor. Replicates were achieved by repeating the experiments with a new infection of B-cells from a different donor. For the initial infections for RNA-seq, ATAC-seq, and metabolomics analyses, purified B-cells from two individual donors were independently infected as described above. At each collection time point, cells were divided into appropriate aliquots for RNA-seq, ATAC-seq, and metabolomic profiling, allowing the generation of a cohesive dataset in which all data is derived from the same set of cells.

### RNA-seq

Total RNA was isolated from 1.5 x 10^6^ cells using the Direct-zol microprep kit following the manufacturer’s protocol (Zymo Research). RNA samples were submitted to the Wistar Institute genomics core facility for initial analysis of RNA quality, with each sample having a RIN value greater than 8.5 (TapeStation, Agilent Technologies). Sequencing library preparation was then completed using the QuantSeq 3’-mRNA kit (Lexogen) to generate Illumina-compatible sequencing libraries according to the manufacturer’s instructions. Sequencing was done with an Illumina NextSeq500 on high output mode to generate ~4x10^8^ 1 x 75bp reads across 8 multiplexed and pooled samples.

### ATAC-seq

The ATAC-seq protocol was done mostly as described previously [29]. Briefly, 5 x 10^4^ cells were washed in 50μl cold PBS, spun down at 500 x g and 4°C for 5mins, and resuspended in 50μl cold lysis buffer (10mM Tris pH 7.5, 10mM NaCl, 3mM MgCl_2_, 0.1% IGEPAL CA-630). Resuspended cells were immediately spun at 500 x g and 4°C for 10mins, and the supernatant was removed. The pellet was then resuspended in a 50μl Tn5 transposase reaction following the manufacturer’s protocol (Nextera Tn5 transposase kit, Illumina) and incubated in a thermomixer at 37°C and 300rpm for 30mins. DNA was purified using a MinElute PCR purification kit (Qiagen) and eluted in 10μl to be used in library amplification. PCR amplification of tagmented DNA was done using the NEBNext HiFi PCR mastermix (New England Bioloabs) with a universal forward and sample-specific reverse oligo for sample barcoding using the following cycling parameters: initial incubations of 72°C for 5 mins and 98°C for 30sec, followed by cycling of 98°C for 10sec, 63°C for 30sec, and 72°C for 1min. The number of cycles was determined empirically for each sample using an aliquot of the PCR as template for an additional qPCR to determine the number of cycles that achieves 1/3 max fluorescence to ensure that libraries do not reach saturation. Total cycle numbers ranged from 10 to 15 cycles. PCR products were run on a 1% agarose gel, regions from ~50bp to ~1kb were excised, and DNA was extracted using a gel extraction kit (Qiagen). Purified DNA was submitted to the Wistar Institute Genomics core facility for quality analysis (2100 Bioanalyzer, Agilent Technologies) and sequencing. All samples were sequenced twice on an Illumina NextSeq500 on high output mode to generate a total of ~8x10^8^ 1 x 75bp reads across 8 multiplexed and pooled samples.

### Mass spectrometry analysis

Polar metabolites were extracted from 1.5x10^6^ cells for each sample using 80% methanol, and targeted quantitation of metabolites was performed on a SCIEX 5500 QTRAP triple quadrupole mass spectrometer equipped with a Turbo V source and coupled to a Shimadzu Nexera UHPLC system. Samples were injected onto a ZIC-pHILIC 2.1-mm i.d × 150 mm column (EMD Millipore). Buffer A was 20 mM ammonium carbonate, 0.1% ammonium hydroxide and buffer B was acetonitrile. The chromatographic gradient was run at a flow rate of 0.15 ml/min for 80–20% B over 20 min, 20–80% B over 0.5 min, and hold at 80% B for 7.5 min. Metabolites were targeted using selected reaction monitoring in positive and negative ion modes. Peak areas from the total ion current for each metabolite-selected reaction monitoring transition were integrated using the MultiQuant v3.0.2 software (SCIEX).

### RT-qPCR for ADA

To assess ADA expression levels, Trizol (Invitrogen) was used to isolate total RNA from primary B-cells infected with EBV, EBV^+^, or EBV^-^ cell lines. Reverse transcription was carried out on equal amounts of DNase-treated RNA using SuperScriptIV reverse transcriptase (Invitrogen), random priming mix (New England Biolabs), and RNase inhibitor (New England Biolabs) following the manufacturer’s instructions. qPCR was performed with Power SYBR green 2X PCR mastermix with primers for ADA (S1 Table). Data was normalized to actin (S1 Table) or GUSB (S1 Table) as indicated. Statistical significance of mean differences in normalized ADA levels was determined by two-tailed *t*-test.

### shRNA knockdown

Lentiviruses for delivery of shRNAs were produced by co-transfecting pLKO.1-based shRNA expression plasmids with the packaging plasmids pMD2.G and pSPAX2 in HEK293T cells at a ratio of 4:3:1 (shRNA:pSPAX2:pMD2.G) with lipofectamine 2000 (Invitrogen). Four independent shRNA plasmids against ADA (TRCN0000051483–86) were supplied by the Wistar Institute High-throughput Screening Facility. Medium was changed at 24hr post-transfection, and supernatants were collected at 48hrs post-transfection, fresh medium was added, and supernatants were collected again at 48hrs post-transfection. Supernatants were then spun at 1000 x g for 15mins to remove cellular debris, and then passed through a 0.45μM filter.

To knockdown expression of ADA, 5x10^6^ BJAB, Mutu I, or LCL cells were resuspended in 5ml lentivirus-containing supernatant and spun at 450 x g for 90mins with 8μg/ml polybrene (Santa Cruz Biotechnology). Cells were then resuspended and maintained in normal medium for 48hrs at which point cells were switched to puromycin-containing medium. Due to their differing sensitivity to puromycin, as empirically determined with non-lentivirus-transduced cells, BJAB and Mutu I cells were maintained in 1μg/mg puromycin, while LCLs were maintained in 0.25μg/ml. Cell proliferation was measured by counting live cells, stained with trypan blue, and quantitating as a percentage of shControl-transduced cell growth. Statistically significant differences in proliferation percentages were determined by two-tailed *t*-test.

To knockdown expression of ADA in primary B-cells, 5x10^6^ B-cells were resuspended in 5ml lentivirus-containing supernatant and spun at 450 x g for 90 min with 8 μg/ml polybrene. Cells were resuspended in B-cell culture medium containing 200U/ml IL-4 and 500 ng/ml CD40L (Peprotech, Cranbury, NJ), then seeded in a 96-well plate. Cell viability was determined using the Cell Titer-Glo assay (Promega) at indicated time.

### Pharmacological ADA inhibition

Sensitivity to pharmacological inhibition of ADA in multiple EBV^+^ and EBV^-^ cell lines was determined by seeding cells in triplicate in a 384 well plate format at a concentration of 1000 cells per well 24hrs prior to treatment. Cells were then treated for 72hrs with a serial dilution of *erythro*-9-Amino-β-hexyl-α-methyl-9H-purine-9-ethanol hydrochloride (EHNA, Sigma) or pentostatin (Sigma) over a 10-point concentration range with two-fold dilutions from 3mM to 3μM. Cell viability was determined using the Cell Titer-Glo assay (Promega) and IC_50_ values were calculated. Bortezomib (Sigma) was used as a positive control for cytotoxicity.

### Bioinformatics

#### ATAC-seq

ATAC-seq data was aligned using *bowtie [64]* against the hg19 version of the human genome, followed by *HOMER* [65] to generate bigwig files and call significant peaks using the “-style histone” and “-style factor” options. 364,021 unique ATAC-seq sites with at least one peak that passed an FDR < 5% threshold in at least one sample were considered significant and were included in additional filtering. Normalized signals for significant sites were derived from bigwig files using *bigWigAverageOverBed* tool from the UCSC toolbox [66] with mean0 option and quantile normalized. 25,806 high-confidence sites that showed a normalized signal of at least 4-fold over average global background level (0.4 reads/bp/10M sequenced reads) were taken for further analysis. Significance of pair-wise differences between time points was estimated using unpaired t-test with Benjamin-Hochberg correction for multiple testing, and 8,860 sites that passed an FDR < 5% threshold were considered significant and used for unsupervised clustering. Clustering was performed on z-score scaled data using a k-means algorithm with standardized Euclidean distance. Mean cluster profiles were then used for additional hierarchical clustering to show cluster relationships. Motif analysis for all high-confidence ATAC-seq sites as well as for regions from each cluster was performed using *HOMER* [65] with default options.

#### ChIP-seq

Existing ChIP-seq data for various transcription factors and histone marks was downloaded from ENCODE [67] for the GM12878 cell line or the GEO database (GSE73887 for EBNA1, GSE47629 for EBNA2/EBNA3C) and was aligned using *bowtie* [64] against the hg19 version of the human genome. *HOMER* was used to generate normalized bigwig files [65]. Three kilobase regions on either side of the center of ATAC-seq sites were used to determine average ChIP-seq signal derived from bigwig files using 60bp bins and the *bigWigAverageOverBed* tool from the UCSC toolbox [66] with mean0 option. Average profiles for ChIP-seq data were calculated for each cluster of ATAC-seq regions and visualized. Heatmap of ChIP-seq data was generated using average values for bins with top 10^th^ quantile signal across all regions.

### RNA-seq

RNA-seq data was aligned using the bowtie2 [64] algorithm against the hg19 human genome version, followed by RSEM (v1.2.12) software [68] to estimate read counts and RPKM values using gene information from Ensemble transcriptome version GRCh37.p13. DESeq2 [69] was used on raw counts to estimate significance of expression differences between any two experimental groups and generate normalized counts that were z-score scaled for clustering. Overall gene expression changes were considered significant with an FDR < 5%. Gene set enrichment analysis was done using QIAGEN's Ingenuity Pathway Analysis software (IPA, QIAGEN Redwood City,www.qiagen.com/ingenuity) using the “Canonical pathways” option. Clustering of RNA-seq data was performed in the same way as ATAC-seq data. Pearson correlation coefficient was calculated between expression of genes and every high-confidence ATAC-seq site that was within 1kb from the gene TSS, or within a distant enhancer of the gene as supported by the Genehancer database [33] and correlations with r > 0.5 were considered.

### Metabolomics

Analyses of metabolomic data alone and integrated metabolomic and transcriptome data was done using the MetaboAnalyst [42, 43]. Briefly, DESeq2 [69] was used on raw counts of metabolite samples to determine significant changes in metabolite levels. Metabolites with significantly altered levels at the specified time point compared to Day 0 (|FC| > 2, FDR < 5%) were used in the MetaboAnalyst Enrichment Analysis module using the Small Molecule Pathway Database [70] against a background reference of all the metabolites targeted in the dataset. For the integrated analysis of metabolomic and transcriptomic data, both significantly altered metabolites and genes (|FC| > 2, FDR < 5%) were input in the MetaboAnalyst Joint Pathway Analysis module using the Kyoto Encyclopedia of Genes and Genomes (KEGG, database version Oct2019). Only metabolism specific pathways that are made up of both metabolites and metabolism-related genes, were included in the analysis. The impact score for each pathway was measured based on degree centrality, or the number of links connecting nodes in a pathway, so that changes to more central nodes in a pathway have a larger impact than outlying nodes. Pathway change significance was determined with a hypergeometric enrichment test with the gene and metabolites combined into a single query pool.

Data visualization was done using a number of tools including the gplots (v3.0.3)[71], and ggplot2 (v3.3.0)[72], packages for R (v3.5.2, “Eggshell Igloo)[73], as well as Heatmapper[74] and nVenn [75].

## Supporting information

S1 Table Sequences of oligos used in this study. (XLSX)

S2 Table Complete metabolomic profiling dataset. (XLSX)

S1 Fig Increased CTCFL (BORIS) expression correlates with altered chromatin accessibility. (A-C) Breakdown of integrated dataset for representative genes associated with BORIS binding sites. Relative expression level and DNA accessibility at each timepoint are shown (A), along with examples of DNA binding for BORIS and CTCF at associated ATAC-seq peaks for FOS (B) and JUN (C). Normalized expression levels for both *CTCFL* (D) and *CTCF* (E) are shown across the experimental time course. (F) Western blot analysis of CTCFL and GAPDH from EBV infected B-cells at times indicated.
(TIF)

S2 Fig Additional EBV-mediated changes the B-cell transcriptome. (A-B) Relative expression levels for interferon inducible genes (A) and multiple cellular transcription factors (B). (C-E) Volcano plots showing differentially expressed genes compared to Day 0 for Day 2 (C), Day 7 (D), and Day 21 (E).
(TIF)

S3 Fig Integration of ATAC-seq and RNA-seq in directly correlated genes. The ATAC-seq peaks associated with the subset of directly correlated genes were broken down by distribution among RNA-seq clusters. Overall breakdown of distribution of directly correlated genes for both ATAC-seq peak clusters and RNA-seq peak clusters is also included.
(TIF)

S4 Fig EBNA enrichment at TSS and enhancer regions in directly correlated genes. Binding sites for EBNA proteins occur more often than expected due to chance at both the TSS and enhancer region for the directly correlated genes, implying enriched binding of EBNA proteins among this gene subset. Enrichment was calculated as percent of genes with EBNA at a chromatin site correlated with gene expression versus percent of genes with EBNA at all sites without considering the correlation.
(TIF)

S5 Fig Enrichment analysis of significantly changed metabolites. (A-C) Metabolic pathways altered during the time course of EBV-mediated B-cell immortalization using only significantly changed metabolites (|FC| > 2, FDR < 5%) at Day 2 (A), Day 7 (B), and Day 21 (C).
(TIF)

S6 Fig EBNA1 activation of ADA in 293T cells. EBNA1 was expressed by transient transfection with pCMV-3xFLAG-EBNA for 3 days followed by RT-qPCR for ADA mRNA relative to GAPDH. Western blot for EBNA1 expression (right panel). *, p<0.05; p values determined by two-tailed t-test; data represents 3 independent experiments.
(TIF)

S7 Fig shADA knockdown in Mutu I and primary B-cells treated with IL4/CD40L. (A) Mutu I cells were transduced with lentivirus shADA or shControl, and relative proliferation was determined by Cell Titer-Glo assay and normalized to shControl-transduced cells (top panel) and Western blot for ADA and GAPDH (lower panels). (B) Primary B-cells were transduced with shADA or shControl lentivirus then treated with IL4/CD40 ligand and assayed 7 days post- transduction/treatment by Cell Titer-Glo (top panel) or RT-qPCR for ADA relative to GUSB mRNA. ***, p < .001, **, p < .01, *, p<0.05; p values determined by two-tailed t-test; data represents 3 independent experiments.
(TIF)

S8 Fig ADA inhibitors dose response curves for LCL, MutuI, and HK1 cells. LCL352, MutuI, or HK1 were incubated with either EHNA or pentostatin a 10-point concentration range with twofold dilutions (3.9 μM to 2mM) from 0.1 to 10 μM for 3 days and then assayed by Cell Titer-Glo. IC_50_ values were determined using PRISM software.
(TIF)

S9 Fig EHNA inhibits EBV-induced B-cell immortalization and cell-size expansion. (A) Primary B-cells were infected with EBV or mock infection or IL4/CD40L treatment, and then incubated with EHNA at concentrations ranging from 0.1 μM to 1 mM. Cells were then assayed at 2, 7, 14, and 21 days and assayed by Cell Titer-Go. (B and C) Primary B-cells treated with EBV or Mock or IL-4/CD40L were treated with EHNA at 1, 10, 100 μM, or 1 mM and assayed at day 2 for cell size by forward scatter using flow cytometry (C) histogram of “large-cell” population.
(TIF)
