# Supplementary figures and images for: A multi-omics approach to Epstein-Barr virus immortalization of B-cells reveals EBNA1 chromatin pioneering activities targeting nucleotide metabolism
Source: PLoS Pathog. 2021 Jan 26;17(1):e1009208. doi: 10.1371/journal.ppat.1009208 (PMC7864721; doi:10.1371/journal.ppat.1009208)

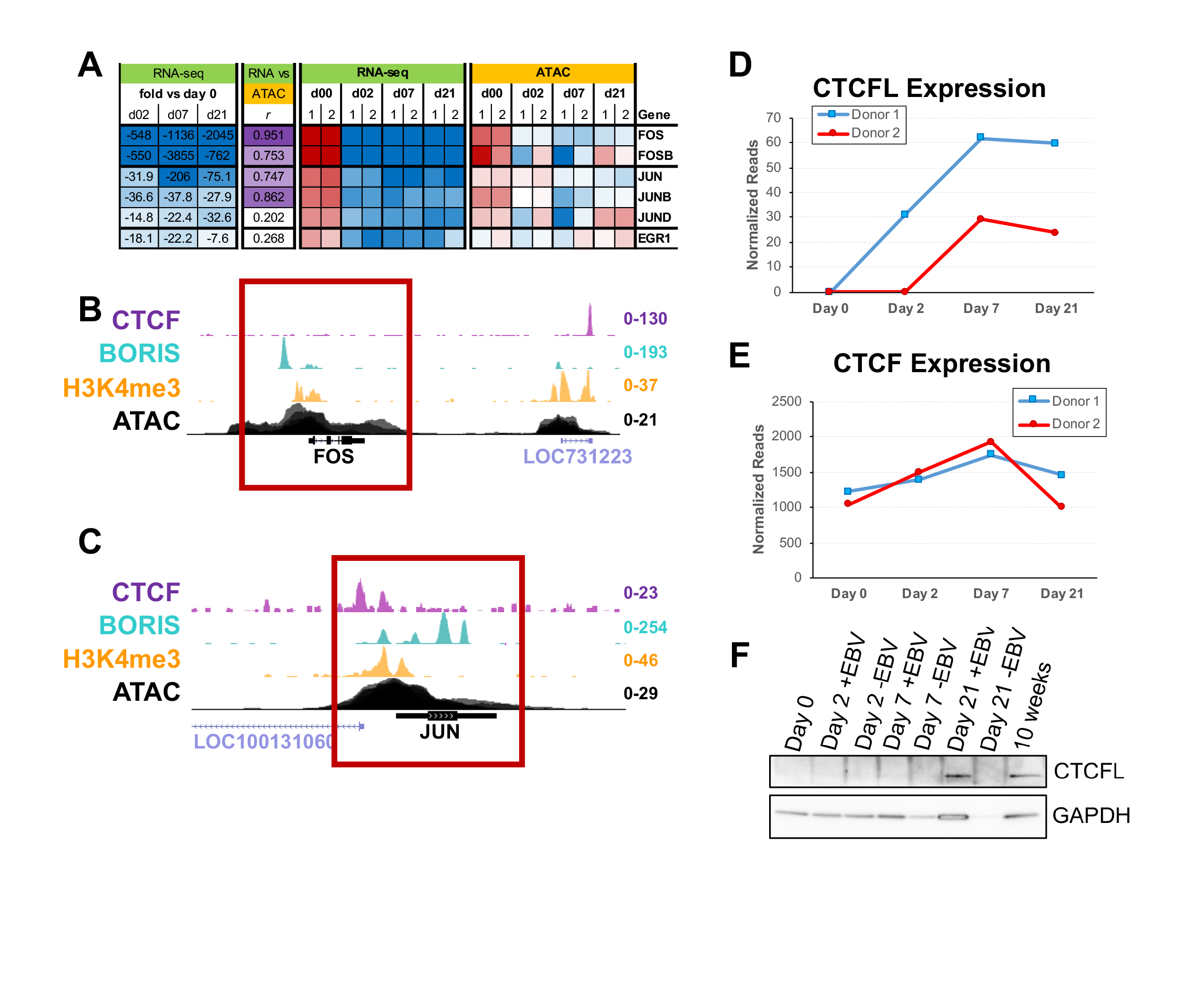

Supplement: S1 Fig — (A-C) Breakdown of integrated dataset for representative genes associated with BORIS binding sites. Relative expression level and DNA accessibility at each timepoint are shown (A), along with examples of DNA binding for BORIS and CTCF at associated ATAC-seq peaks for FOS (B) and JUN (C). Normalized expression levels for both CTCFL (D) and CTCF (E) are shown across the experimental time course. (F) Western blot analysis of CTCFL and GAPDH from EBV infected B-cells at times indicated. (TIF) [file ppat.1009208.s003.tif]

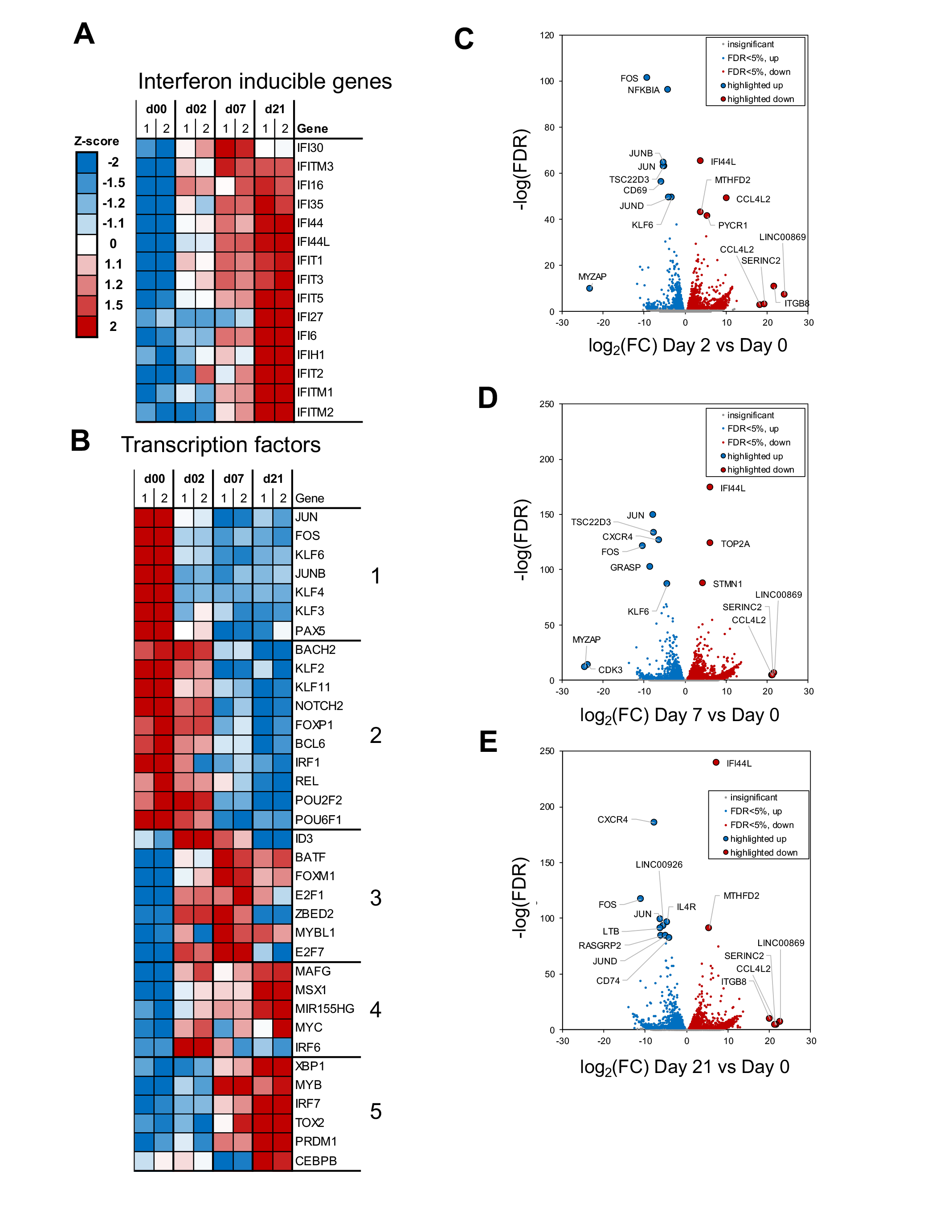

Supplement: S2 Fig — (A-B) Relative expression levels for interferon inducible genes (A) and multiple cellular transcription factors (B). (C-E) Volcano plots showing differentially expressed genes compared to Day 0 for Day 2 (C), Day 7 (D), and Day 21 (E). (TIF) [file ppat.1009208.s004.tif]

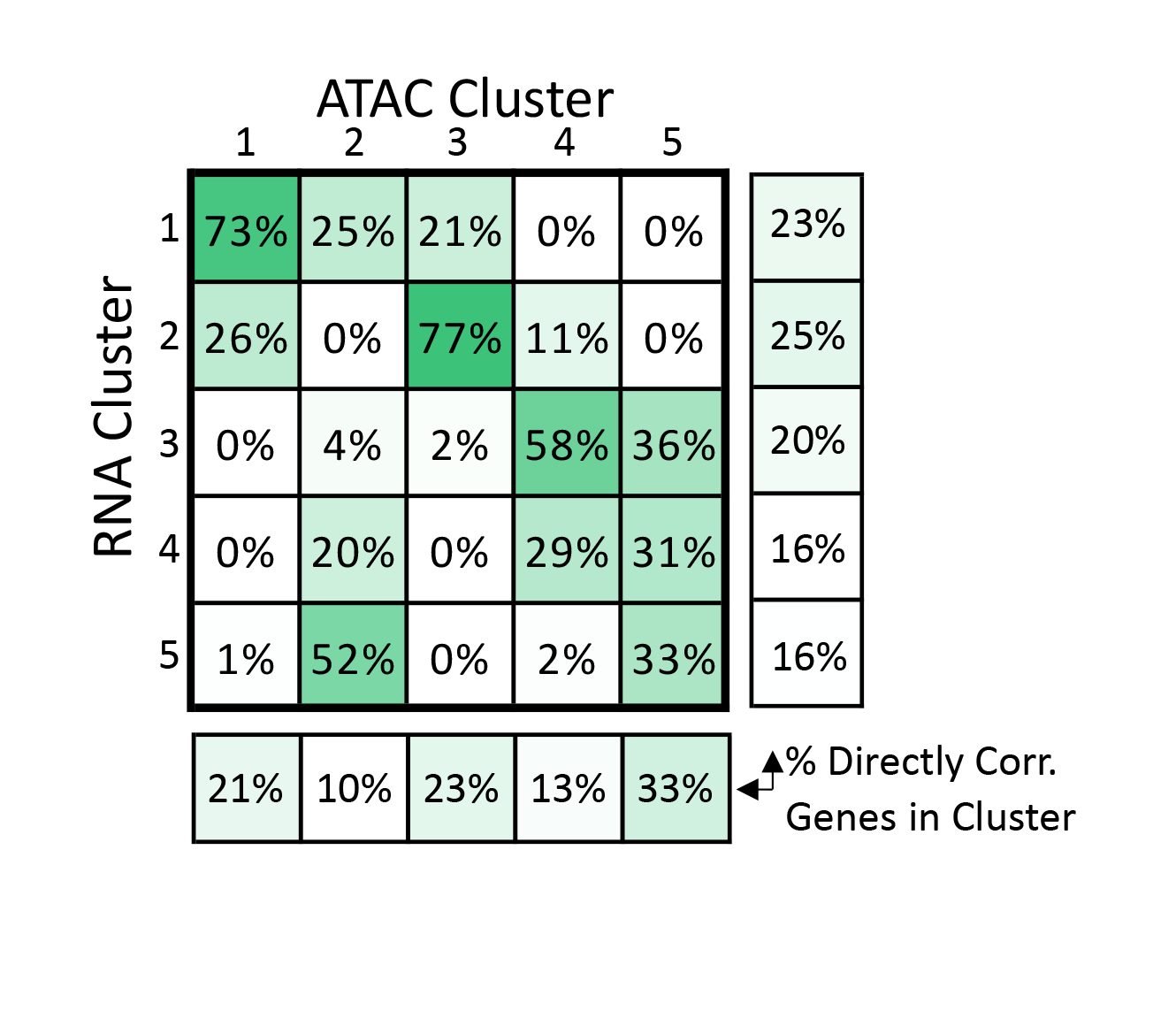

Supplement: S3 Fig — The ATAC-seq peaks associated with the subset of directly correlated genes were broken down by distribution among RNA-seq clusters. Overall breakdown of distribution of directly correlated genes for both ATAC-seq peak clusters and RNA-seq peak clusters is also included. (TIF) [file ppat.1009208.s005.tif]

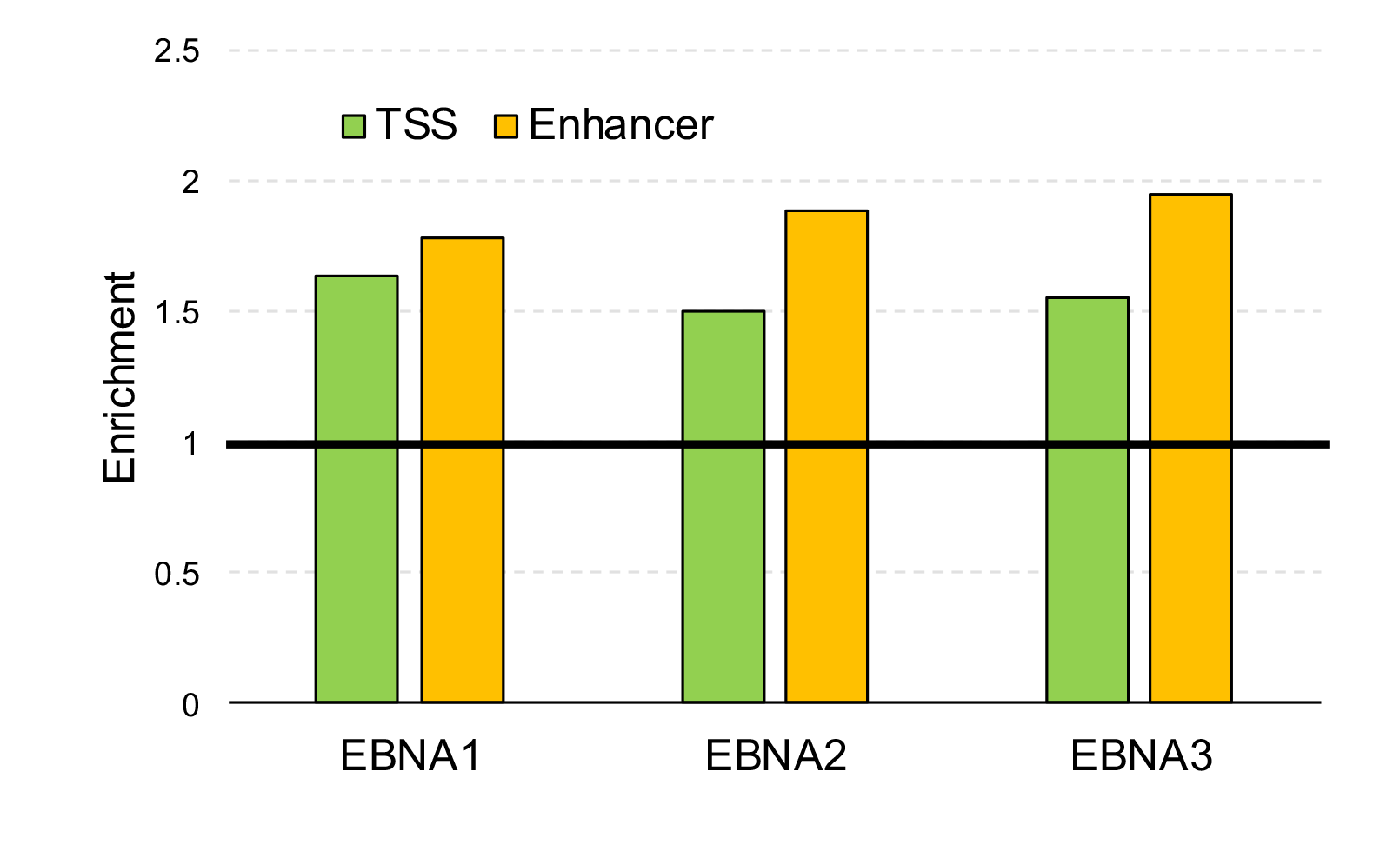

Supplement: S4 Fig — Binding sites for EBNA proteins occur more often than expected due to chance at both the TSS and enhancer region for the directly correlated genes, implying enriched binding of EBNA proteins among this gene subset. Enrichment was calculated as percent of genes with EBNA at a chromatin site correlated with gene expression versus percent of genes with EBNA at all sites without considering the correlation. (TIF) [file ppat.1009208.s006.tif]

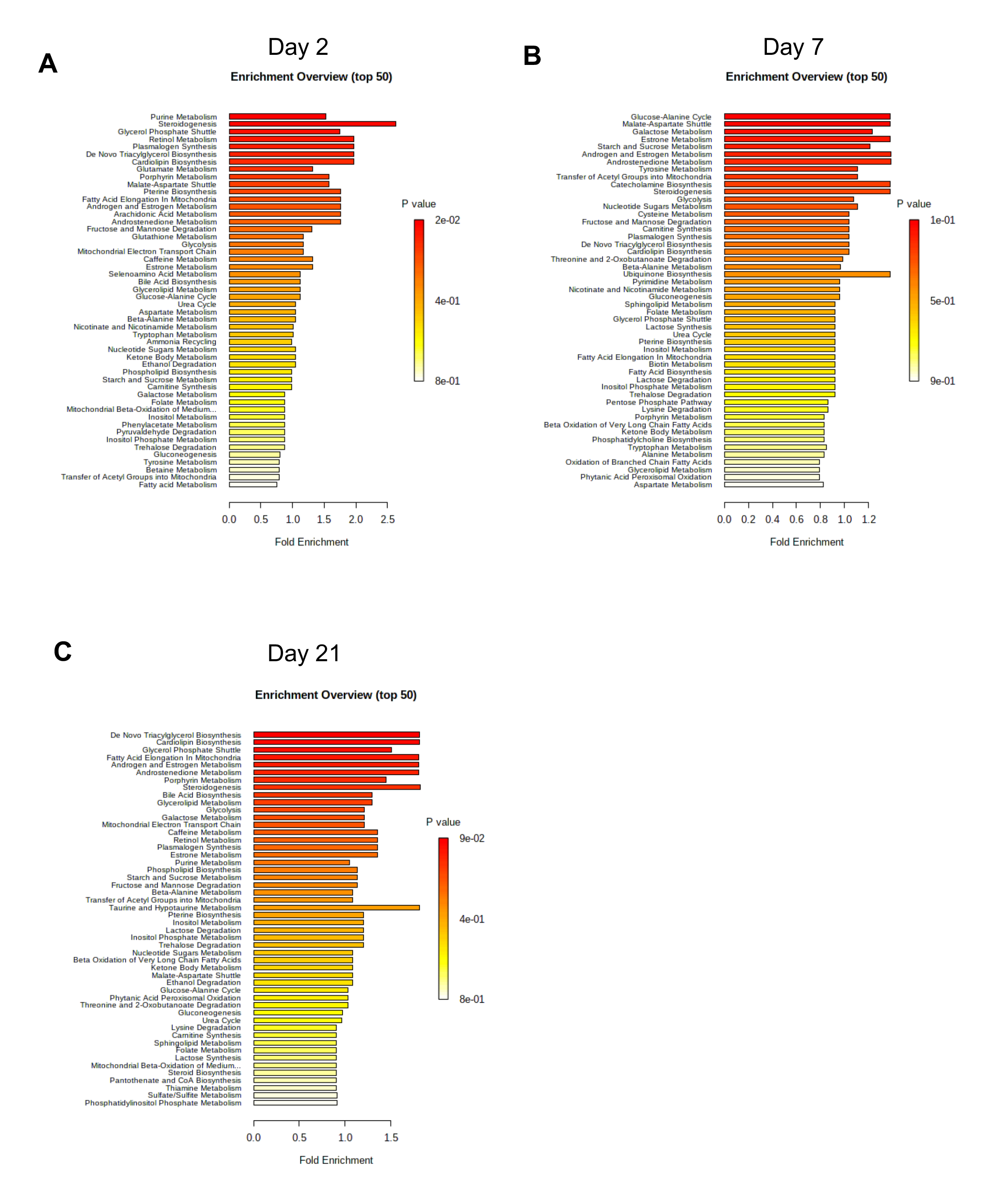

Supplement: S5 Fig — (A-C) Metabolic pathways altered during the time course of EBV-mediated B-cell immortalization using only significantly changed metabolites (|FC| > 2, FDR < 5%) at Day 2 (A), Day 7 (B), and Day 21 (C). (TIF) [file ppat.1009208.s007.tif]

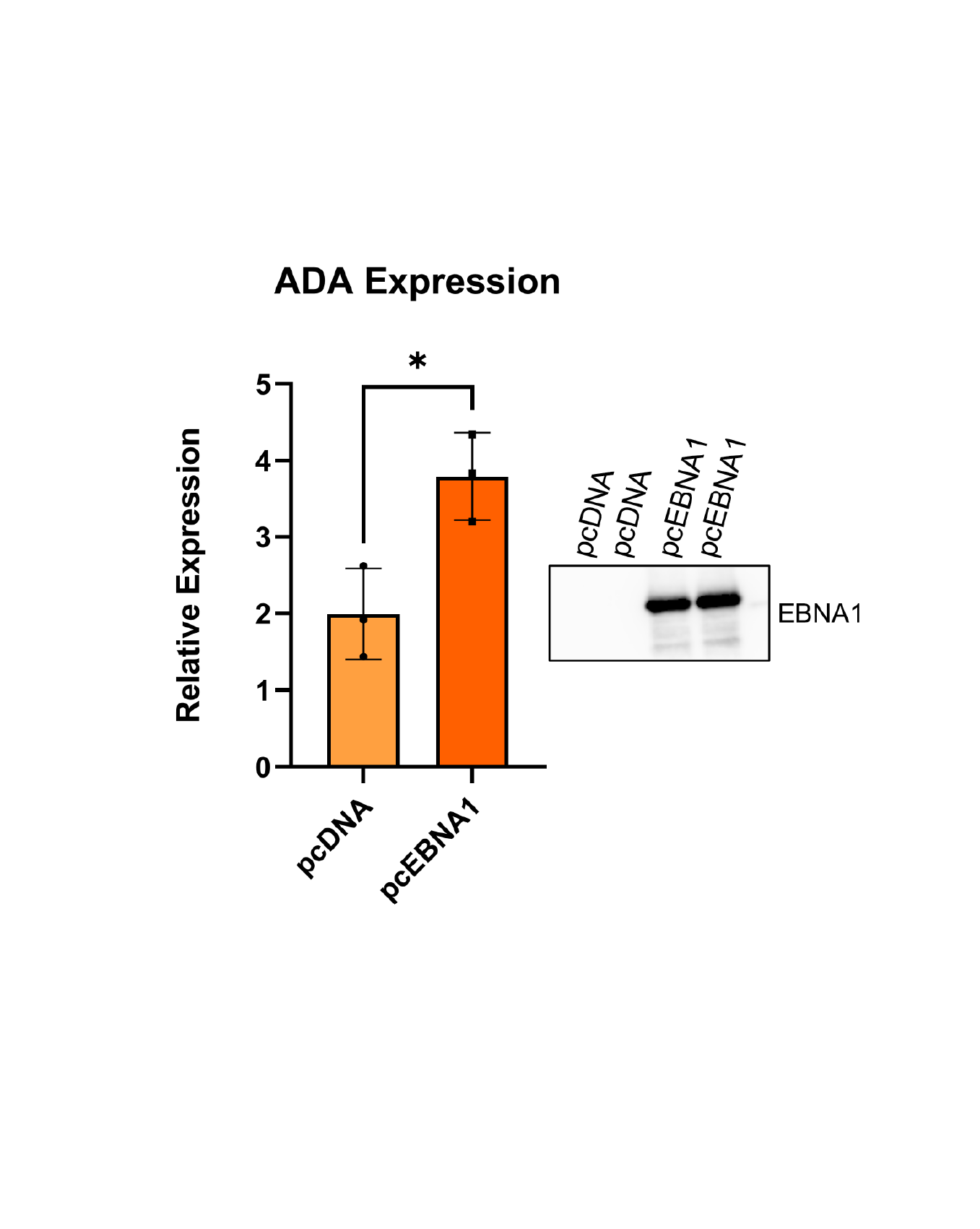

Supplement: S6 Fig — EBNA1 was expressed by transient transfection with pCMV-3xFLAG-EBNA for 3 days followed by RT-qPCR for ADA mRNA relative to GAPDH. Western blot for EBNA1 expression (right panel). *, p<0.05; p values determined by two-tailed t-test; data represents 3 independent experiments. (TIF) [file ppat.1009208.s008.tif]

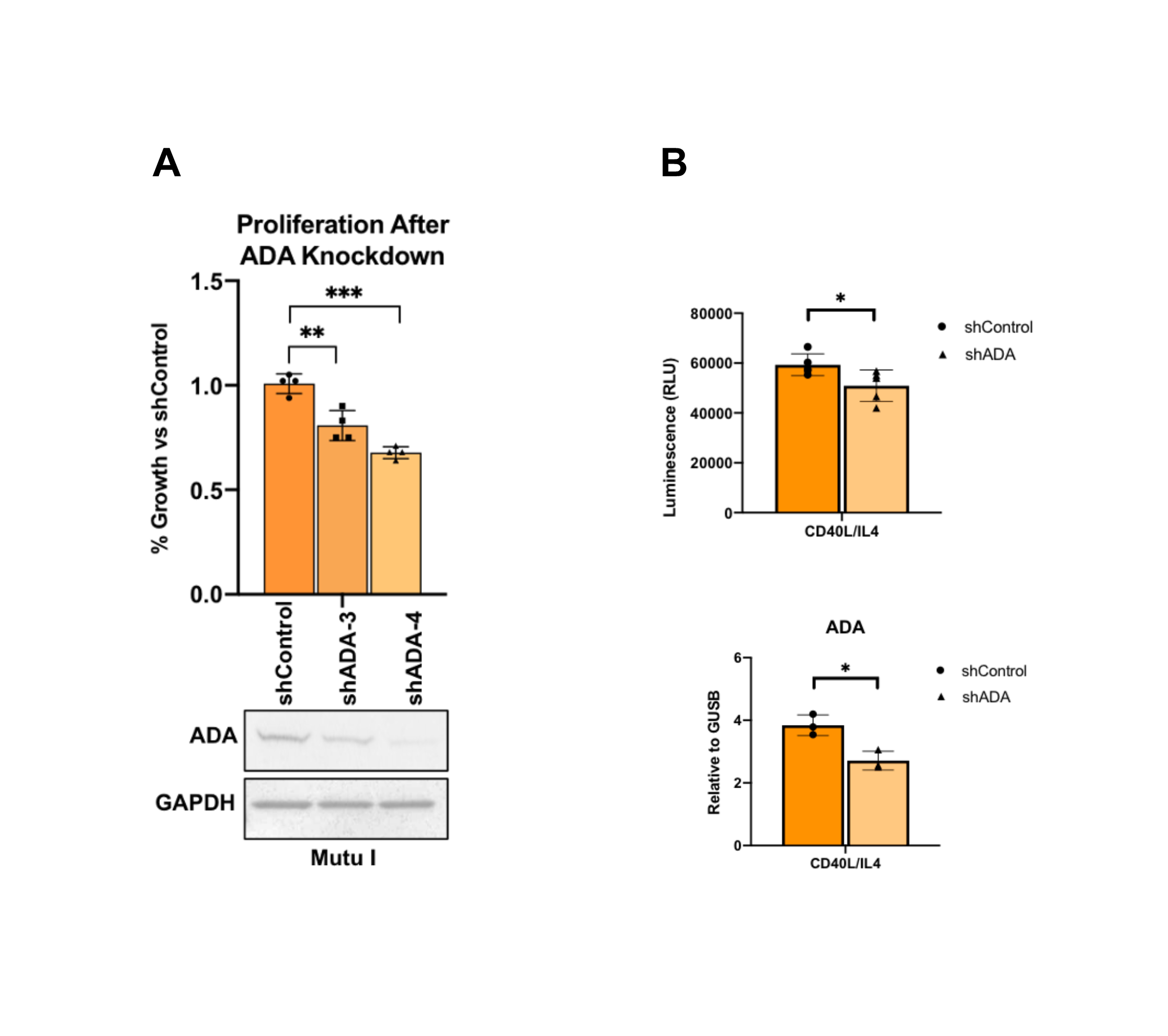

Supplement: S7 Fig — (A) Mutu I cells were transduced with lentivirus shADA or shControl, and relative proliferation was determined by Cell Titer-Glo assay and normalized to shControl-transduced cells (top panel) and Western blot for ADA and GAPDH (lower panels). (B) Primary B-cells were transduced with shADA or shControl lentivirus then treated with IL4/CD40 ligand and assayed 7 days post- transduction/treatment by Cell Titer-Glo (top panel) or RT-qPCR for ADA relative to GUSB mRNA. ***, p < .001, **, p < .01, *, p<0.05; p values determined by two-tailed t-test; data represents 3 independent experiments. (TIF) [file ppat.1009208.s009.tif]

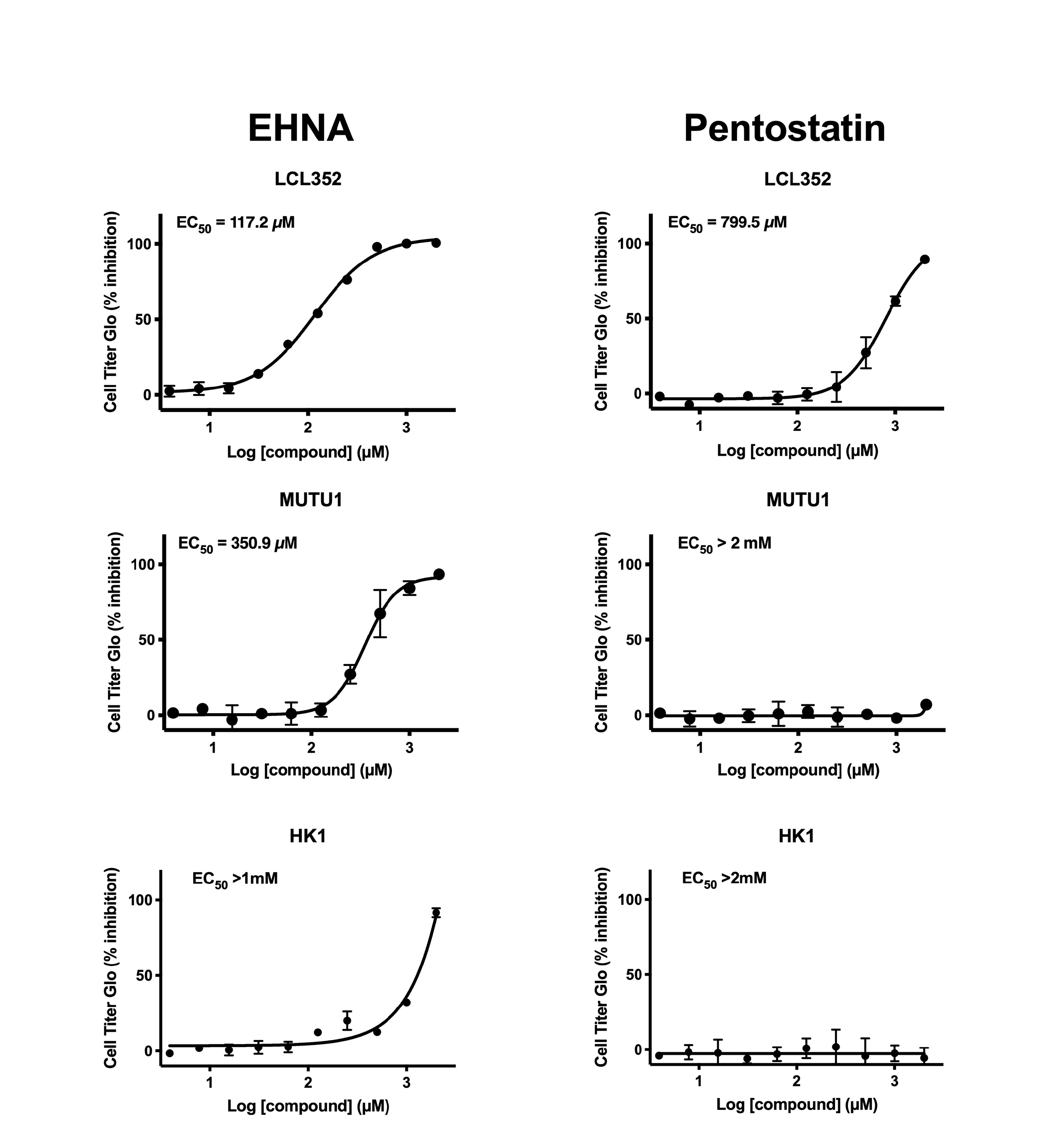

Supplement: S8 Fig — LCL352, MutuI, or HK1 were incubated with either EHNA or pentostatin a 10-point concentration range with twofold dilutions (3.9 μM to 2mM) from 0.1 to 10 μM for 3 days and then assayed by Cell Titer-Glo. IC50 values were determined using PRISM software. (TIF) [file ppat.1009208.s010.tif]

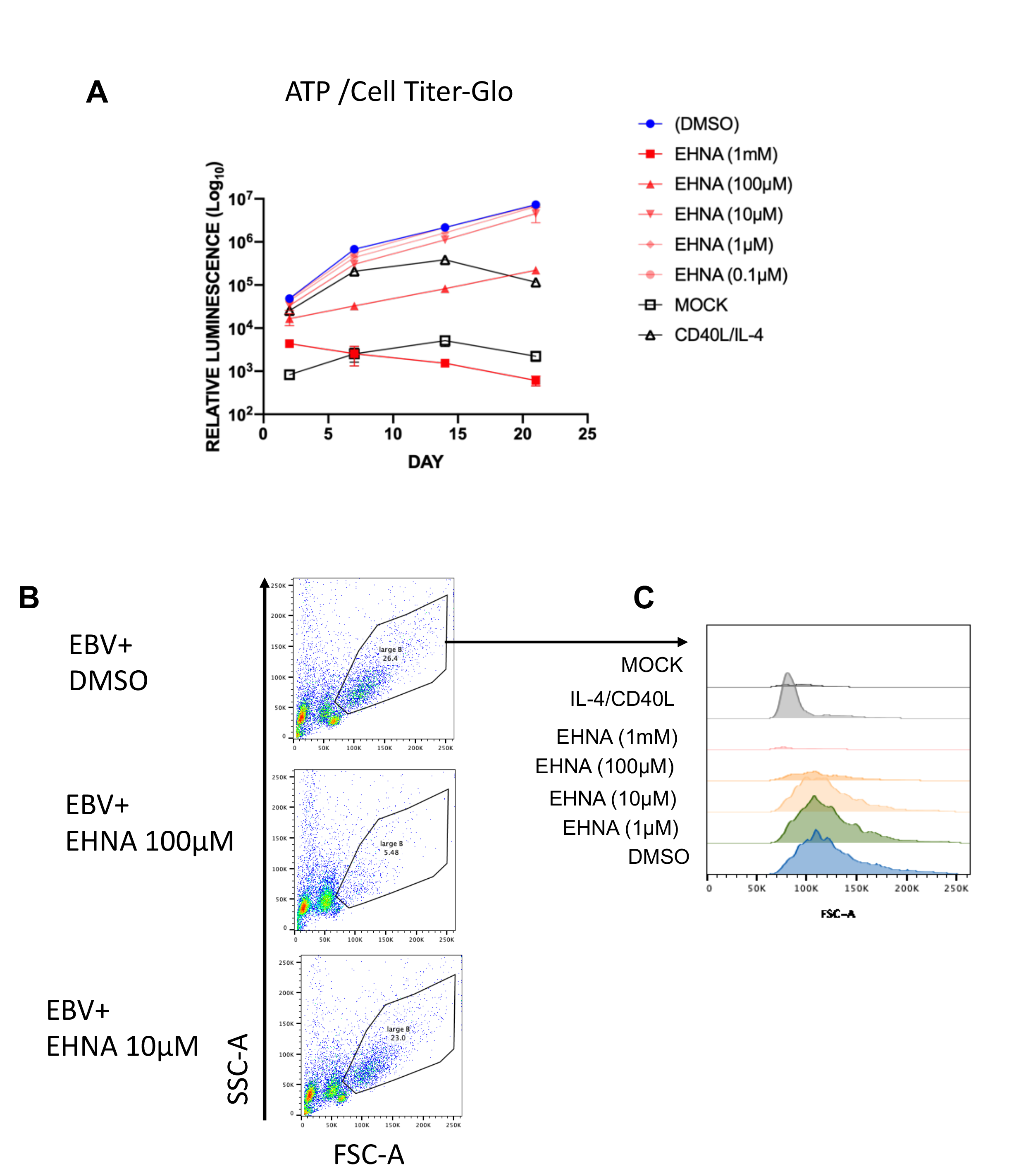

Supplement: S9 Fig — (A) Primary B-cells were infected with EBV or mock infection or IL4/CD40L treatment, and then incubated with EHNA at concentrations ranging from 0.1 μM to 1 mM. Cells were then assayed at 2, 7, 14, and 21 days and assayed by Cell Titer-Go. (B and C) Primary B-cells treated with EBV or Mock or IL-4/CD40L were treated with EHNA at 1, 10, 100 μM, or 1 mM and assayed at day 2 for cell size by forward scatter using flow cytometry (C) histogram of “large-cell” population. (TIF) [file ppat.1009208.s011.tif]
